# Mechanotransduction-enhanced bioconstructs fabricated using a bioink comprising collagen and omega-3 fatty acids for gingival tissue regeneration

**DOI:** 10.7150/thno.114503

**Published:** 2025-05-25

**Authors:** GaEun Heo, Hoon Noh, Dogeon Yoon, SooJung Chae, Hanjun Hwangbo, Ji Hye Park, Won Hee Lim, WonJin Kim, GeunHyung Kim

**Affiliations:** 1 Department of Precision Medicine, Sungkyunkwan University School of Medicine (SKKU-SOM), Suwon 16419, Republic of Korea; 2 Department of Orthodontics, School of Dentistry and Dental Research Institute, Seoul National University, Seoul, Republic of Korea; 3 Burn Institute, Hangang Sacred Heart Hospital, College of Medicine, Hallym University, Seoul, Republic of Korea; 4 Department of Convergence Pharmaceutical Science, Korea University, Sejong, 30019, Republic of Korea; 5 Biomedical Institute for Convergence at SKKU (BICS), Sungkyunkwan University, Suwon 16419, Republic of Korea

**Keywords:** bioprinting, omega-3 fatty acid, mechanotransduction, gingival tissue regeneration

## Abstract

**Rationale:** Tissue engineering through three-dimensional (3D) bioprinting has emerged as a highly promising strategy for creating custom-designed 3D bioconstructs that closely mimic native tissue architecture. However, ongoing advancements in bioink formulation and bioprinting processes are required to achieve precise replication of target tissues. In particular, effective vascularization and extracellular remodeling are essential for successful gingival tissue regeneration.

**Methods:** To achieve this, we propose a cell-laden collagen bioink formulation containing omega-3 fatty acids, eicosapentaenoic acid (EPA) and docosahexaenoic acid (DHA), for gingival tissue regeneration. To enhance the mechanotransduction of human gingival fibroblasts (hGFs) encapsulated in the bioink, we employed a shear-induced bioprinting process to activate key signaling pathways, including mechanosensitive channels, which are involved in gingival tissue regeneration.

**Results:** Bioprinted cell constructs subjected to both biochemical and biophysical cues exhibited promising gene expression profiles related to collagen production and angiogenesis, demonstrating the potential of integrating bioprinting with mechanical and biochemical stimulation for gingival tissue engineering. Furthermore, when hGF-laden bioconstructs containing EPA/DHA were implanted subcutaneously into mice, the formation of blood vessel-like structures was clearly observed at four weeks post-transplantation.

**Conclusion:** These results suggest that the engineered bioconstruct, incorporating EPA/DHA-assisted bioinks and mechanical stimulation, may offer a promising strategy for gingival tissue regeneration and the development of a 3D biomimetic model within an oral organ-on-a-chip system.

## Introduction

Gingival recession can occur due to periodontal disease or trauma. Although orthodontic treatment may help prevent or even improve gingival recession, a surgical approach may be necessary depending on the severity and extent of tissue damage 1,2. Traditionally, free gingival grafts, subepithelial connective tissue grafts, and guided tissue regeneration have been used to treat gingival defects 3,4. However, these techniques have several limitations, such as donor site morbidity, variable clinical outcomes, and reliance on exogenous tissue grafts 5,6. To address these challenges, tissue engineering using bioprinting has emerged as a highly promising approach, offering the ability to fabricate customized three-dimensional (3D) cell constructs that replicate native gingival tissue architecture 7-9.

Recent advancements in gingival tissue engineering have focused on leveraging 3D bioprinting, cell-based therapies, and growth factor delivery systems to enhance tissue regeneration 2,10,11. Among these, 3D bioprinting has demonstrated considerable potential by facilitating the precise construction of cell-laden biomaterials, such as collagen-based bioinks, which provide structural and biochemical support to embedded cells 12,13. In particular, human gingival fibroblasts (hGFs), play an important role in promoting gingival tissue formation owing to their ability to produce extracellular matrix (ECM) components and modulate tissue remodeling 14,15. Additionally, bioinks incorporating growth factors, such as bone morphogenetic proteins (BMPs) and vascular endothelial growth factor (VEGF), have been utilized to enhance cellular differentiation and vascularization 16-18. Despite these advances, key challenges remain, including inadequate vascularization and insufficient extracellular remodeling by cells 10,19. In general, the efficient ECM remodeling by hGFs can be an essential factor in gingival tissue regeneration, as it facilitates restoration of native tissue architecture and enhances functional outcomes 10,20,21. In this aspect, appropriate mechanical stimulation of hGFs can contribute to improved ECM organization and mechanical properties of regenerated tissue, such as elasticity and strength 20-24. Furthermore, stimulated fibroblasts exhibit enhanced migratory, proliferative, and matrix-synthetic activities, which are essential for effective tissue remodeling and regeneration 20-24. Therefore, favorable mechanotransduction, that is, translation of mechanical forces into biochemical signals that regulate cellular behaviors, including collagen production, wound healing, and maintenance of tissue integrity, is necessary for achieving functional integration and restoring physiological properties in regenerated gingival tissues.

In this study, we propose a new approach for gingival tissue regeneration using bioprinted cell constructs composed of a bioink containing collagen type I, hGFs, and omega-3 polyunsaturated fatty acids (eicosapentaenoic acid (EPA) and docosahexaenoic acid (DHA)). Porcine skin-derived collagen type I was selected owing to its biocompatibility, ability to support hGF proliferation, and to induce the expression of crucial gingival tissue markers, including collagen types I (Col-I), III (Col-III), and V (Col-V). EPA and DHA were incorporated for their well-documented angiogenic and proliferative effects, as well as their role in enhancing collagen production by modulating fibroblast activities 25-27. Further, these fatty acids exert their effects by modulating critical biochemical pathways, such as the activation of peroxisome proliferator-activated receptor gamma, which regulates inflammation and tissue repair 28,29.

To further enhance mechanotransduction, we utilized a comb-attached bioprinter that applied mechanical stimulation during the bioprinting process. This process facilitates the activation of several signaling pathways, such as PIEZO-1 and TRPV2 that regulate mechanosensitive responses such as proliferation and migration, YAP/TAZ in the Hippo pathway that promotes cell growth and tissue formation, and Wnt signaling that modulates fibroblast differentiation and ECM production 30,31. By examining these pathways and their activation mechanism, we aimed to elucidate the role of mechanotransduction in gingival tissue regeneration and validate the efficacy of bioprinted constructs in promoting tissue repair. The *in vitro* gene expression profiles and structural organization observed in this study demonstrate the potential of integrating modified bioprinting with biochemical cues. Additionally, *in vivo* subcutaneous experiments demonstrated the formation of blood vessel-like structures. Based on the obtained results, this approach produces bioconstructs with activated cellular mechanosensitive pathways and improved vascularization, offering an efficient strategy to advance gingival tissue engineering.

## Results and discussion

### Comparative evaluation of gingival decellularized ECM and collagen bioinks

Gingival decellularized extracellular matrix (GdECM) is hypothesized to enhance hGF activity owing to its tissue-specific composition and the presence of native bioactive components 32. However, the extraction and decellularization of porcine gingival tissue are labor-intensive and yield very low quantities of usable material. As a more accessible alternative, skin-derived collagen type I (Col), which is relatively easier to extract and process, was selected for comparison as a potential bioink in place of GdECM to regenerate gingival tissue in the present study.

**Figure 1:**
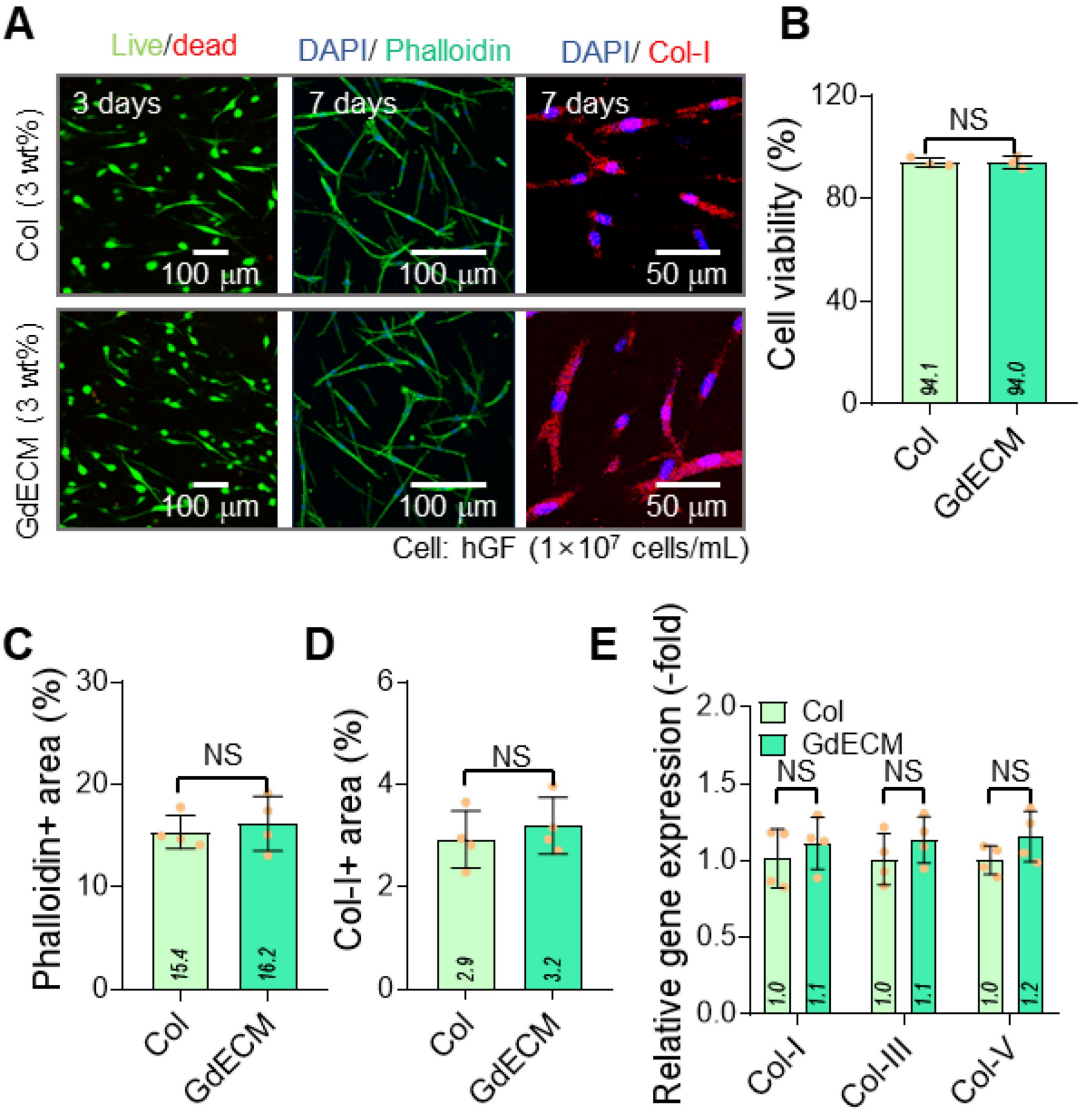
** Comparative evaluation of GdECM and Col bioinks for gingival tissue regeneration.** (A) Live/dead staining images at 3 d and DAPI/phalloidin and DAPI/Col-I staining at 7 d show cell viability exceeding 90% with both bioinks. (B) Quantitative analysis confirms no significant differences in cell viability between the GdECM and Col bioinks (n = 4). (C) Phalloidin-positive cytoskeletal areas and (D) collagen type I distribution are comparable in both groups (n = 4). (E) Gene expression of ECM components, including Col-I, Col-III, and Col-V, demonstrates no statistically significant differences between bioinks (n = 4). All data are mean ± standard deviation (SD) (NS = no significance, analyzed using Student's t-test).

The two bioinks, GdECM (3 wt%) and Col (3 wt%), were compared for their cellular activities with hGFs laden at a density of 1 × 10⁷ cells/mL. Each bioink was printed using a normal bioprinter under the specified printing conditions (nozzle moving speed = 20 mm/s, pneumatic pressure = 170 kPa, barrel/nozzle temperature = 25 °C, and printing plate temperature = 35-38 °C). Figure 1A shows live (green)/dead (red) cells at 3 d and DAPI (blue)/phalloidin (green) and DAPI/collagen-I (Col-I, red) staining at 7 d. The cell viability exceeded 90% in both printed structures using the GdECM and Col bioinks (Figure 1B). Further, phalloidin-positive areas that indicate cytoskeletal organization and collagen type I distribution and reflect matrix integration, showed no statistically significant differences between the two groups (Figure 1C-D). Further, gene expression analyses of ECM components, including collagen types I, III, and V, demonstrated similar expression levels in both bioink groups (Figure 1E). GdECM is considered a promising candidate owing to its tissue-specific composition, which includes various native growth factors, glycosaminoglycans (GAGs), and structural proteins that mimic the *in vivo* gingival microenvironment (Figure S1). Despite the advantages of GdECM, the comparable outcomes observed with the two bioinks suggest that Col can provide a sufficiently supportive microenvironment for hGF activity. The minimal biological difference between the GdECM and Col bioinks may be attributed to the decellularization process used during GdECM preparation, which could have excessively reduced its bioactive properties. Previous studies have also highlighted the challenges in decellularizing dense tissues such as the gingiva, often resulting in the partial loss of key bioactive components such as the proteins essential for cell-matrix interactions 33. Owing to its abundance and ease of bioprinting, we selected the Col bioink, which offers a viable alternative to GdECM as a bioink for gingival tissue engineering 34,35.

### Development of EPA/DHA-containing collagen bioink for gingival tissue regeneration

**Figure 2:**
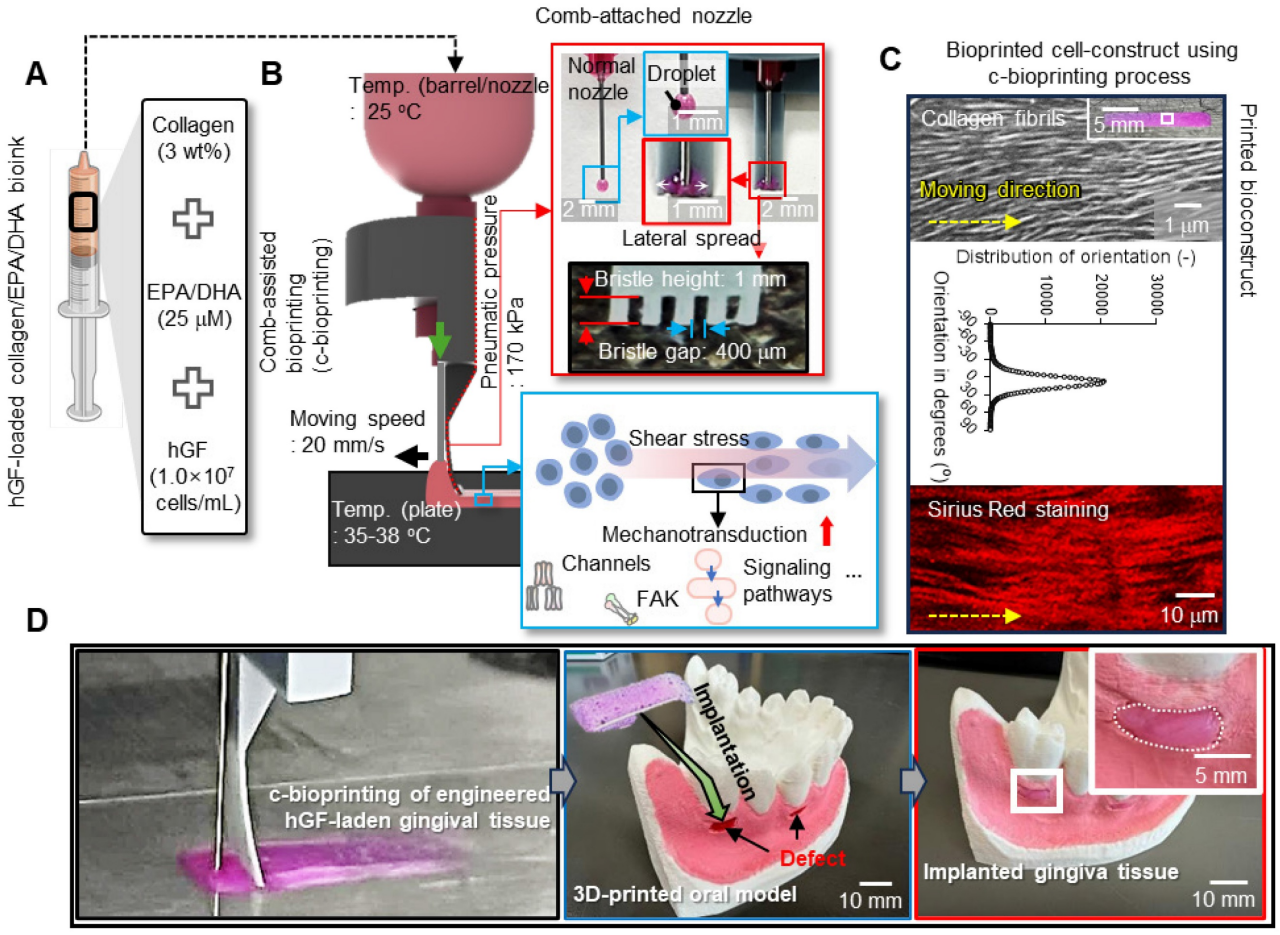
** Development of EPA/DHA-incorporated collagen bioink and c-bioprinting.** (A) Bioink formulation using collagen type I (3 wt%), EPA/DHA (25 μM), and hGFs (1 × 10⁷ cells/mL) to enhance angiogenesis and cellular activity. (B) The comb-assisted bioprinter (c-bioprinter) setup promoting cell alignment through mechanotransduction during printing. (C) Optical, SEM, distribution of collagen fibrils, and Sirius Red staining images confirm structural alignment and organization, mimicking native gingival tissue for enhanced regeneration. (D) Procedure of clinical application for the bioprinted cell-constructs to regenerate gingival tissue.

To achieve enhanced biological outcomes, Col (3 wt%) was selected as the matrix bioink and, as dispersed biological components, EPA and DHA were incorporated into the Col bioink. Eventually, the bioink (ColED) for gingival tissue regeneration consisted of collagen type-I, EPA/DHA, and hGFs (cell density: 1 × 10^7^ cells/mL) (Figure 2A). This bioink, containing appropriately selected concentrations of EPA/DHA, was then utilized in a comb-assisted bioprinter (c-bioprinter) (Figure 2B), which effectively facilitated mechanotransduction to the laden cells by generating additional shear stress through the comb accessory during the printing process. In addition, the c-bioprinter has guided the orientation of collagen fibrils within the bioconstructs (Figure 2C), promoting the alignment of hGF cells to mimic the native organization of gingival tissue, cellular behavior of hGFs, and gingival tissue formation. The bioconstructs were successfully applied to the gingival defects shown in the 3D-printed oral model (Figure 2D). As shown in the images, the bioprinted constructs demonstrated suitable adaptability to gingival defects indicating significant promise for personalized gingival tissue regeneration.

In general, EPA and DHA are well-established bioactive molecules that enhance cellular functions, particularly angiogenesis 36-38. However, their application in tissue engineering can be constrained by concentration-dependent cytotoxicity, where excessive levels adversely affect cell viability 36,39. To determine the appropriate concentration of EPA/DHA for use in the Col-based bioinks intended for gingival tissue regeneration, we assessed their effects on hGFs and angiogenesis in human umbilical vein endothelial cells (HUVECs).

**Figure 3:**
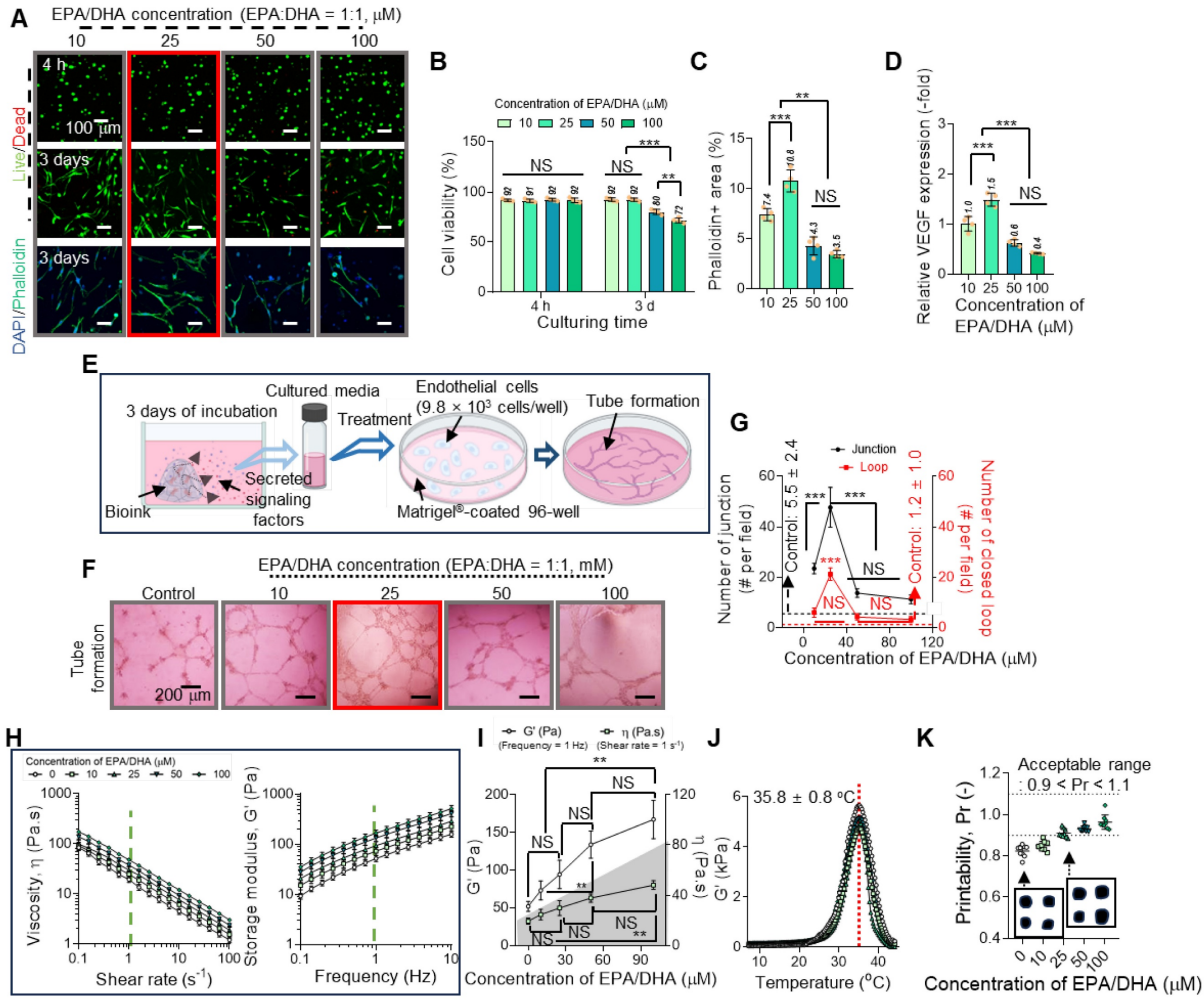
** Concentration-dependent effects of EPA/DHA on hGF viability, cytoskeletal organization, and angiogenesis.** (A) Live/dead staining images at 4 hours and at 3 d, and DAPI/phalloidin staining at 3 d, showing high cell viability and active F-actin formation at 10 and 25 μM, with a decline at concentrations >25 μM (n = 4). (B) Quantitative analysis of cell viability highlights optimal viability at 25 μM EPA/DHA (n = 4). (C) Quantitative analysis of phalloidin-positive areas reveals maximal cytoskeleton formation at 25 μM, with a decline at concentrations > 25 μM (n = 4). (D) VEGF release peaks at 25 μM, indicating enhanced angiogenic signaling at this concentration (n = 4). (E) Schematic of the tube formation assay with HUVECs cultured on Matrigel-coated plates containing EPA/DHA. (F) Optical images of tube formation demonstrate optimal endothelial network formation at 25 μM. (G) Quantitative analysis of junctions and closed loops reveals maximal angiogenesis at 25 μM, with a decline at higher concentrations (n = 4). (H) Shear viscosity (η) vs. shear rate curves and storage modulus (G′) vs. frequency and (I) their values measured at 1 Hz and a shear rate of 1 s^-1^ (n = 3). (J) Comparison of collagen fibrillation temperature for the bioinks (n = 3). (K) Printability for various concentrations of EPA/DHA. All data are mean ± SD (NS = no significance, **p < 0.01, ***p < 0.001, analyzed using ANOVA with Tukey's HSD post-hoc test).

To evaluate the influence of EPA/DHA, concentrations ranging from 10 to 100 μM were incorporated into a 3 wt% Col bioink, and their effects on hGF viability and cytoskeletal organization were analyzed (Figure 3A). The results showed high cell viability at 4 h and 3 d along with active F-actin formation at 3 d at lower concentrations (10 and 25 μM) but a decline at concentrations exceeding 25 μM (Figure 3B-C). To further assess the effects of EPA/DHA concentrations on the accumulation of reactive oxygen species (ROS) and cellular apoptosis, DCF-DA imaging and apoptosis-related gene (*BAX, BAD, AIFM1, CASP3, and CASP6*) expression analyses were conducted on hGFs within collagen bioinks containing various concentrations of EPA/DHA (Figure S2) 40,41. Similar to results of the cell viability, a lower DCF-DA-positive area and reduced expression of apoptosis-related genes were observed at lower concentrations at 3 days, whereas an increase was noted at concentrations exceeding 25 μM (Figure S2B-C). In addition, vascular endothelial growth factor (VEGF), a key angiogenic growth factor, was released, with a peak at 25 μM (Figure 3D), indicating appropriate activation of the signaling pathways promoting angiogenesis at this concentration.

To observe the effect of EPA/DHA concentration on the blood vessel formation ability, tube formation was evaluated using HUVECs cultured on Matrigel-coated plates containing varying concentrations of EPA/DHA (Figure 3E). Optical images showing tube formation are shown in Figure 3F. Through these optical images, the number of junctions and number of closed loops, indicative of endothelial cell network formation, were measured, and the numbers were found to be the highest at 25 μM EPA/DHA in the bioink (Figure 3G). However, as the concentration increased beyond this level, the number of junctions decreased, suggesting a decline in angiogenic efficiency. The results thus demonstrated a biphasic response to EPA/DHA concentrations, with critical threshold for maximizing angiogenic potential without compromising cell viability.

The observed concentration-dependent effects of EPA/DHA aligned with previous studies demonstrating their role in activating angiogenic signaling pathways 42. At optimal levels, EPA/DHA can upregulate VEGF expression through activation of peroxisome proliferator-activated receptors and endothelial nitric oxide synthase, promoting endothelial cell proliferation and migration 43,44. This is consistent with peak VEGF release and enhanced tube formation observed at the EPA/DHA concentration of 25 μM. Conversely, higher concentrations may lead to the excessive production of reactive oxygen species 45, inducing oxidative stress and impairing cellular function. This response emphasizes the importance of maintaining EPA/DHA concentrations within a physiologically optimal range to effectively control their therapeutic potential.

Based on these results, we selected 25 μM as the appropriate concentration of EPA/DHA for incorporation into the Col bioink because, at this concentration, EPA/DHA supported hGF viability and cytoskeletal organization as well as maximized VEGF release and angiogenesis in HUVECs.

To evaluate the effects of fatty acids on rheological properties, shear viscosity (η) vs. shear rate and storage modulus (G′) vs. frequency were measured (Figure 3H). The results revealed that most bioinks containing different concentrations of EPA/DHA exhibited clear shear-thinning behavior, demonstrating that the bioinks could be easily extruded through a microscale nozzle. In addition, an increase in the EPA/DHA concentration gradually enhanced both the viscosity and storage modulus of the bioink (Figure 3I). This phenomenon is consistent with findings reported by several researchers 46,47. Guerrero et al. reported that the rheological characteristics of oil-in-water emulsions (O-W emulsions) were enhanced owing to the development of elastic frameworks by protein molecules encapsulating the oil droplets 46. Similarly, in the EPA/DHA-assisted collagen bioink, collagen molecules acted as emulsifiers, physically encapsulating the EPA/DHA droplets 47,48. Furthermore, the collagen fibrillation temperature of the bioink was measured, and the results indicated that the addition of EPA/DHA did not significantly affect the fibrillation temperature, which remained at 35.8 ± 0.8 °C (Figure 3J).

Finally, the printability of the bioink containing EPA/DHA at 25 µM was assessed (Figure 3K). At this concentration, the bioink (P_r_ = 0.92) demonstrated sufficient stiffness to ensure precise filament deposition while maintaining the resolution and stability of the printed struts, as determined by printability (P_r_ = L^2^/16A, where L and A indicate the perimeter and pore area of the mesh structure 49 ), thereby confirming its suitability for bioprinting applications.

### Fabrication and evaluation of EPA/DHA-loaded bioprinted constructs

**Figure 4:**
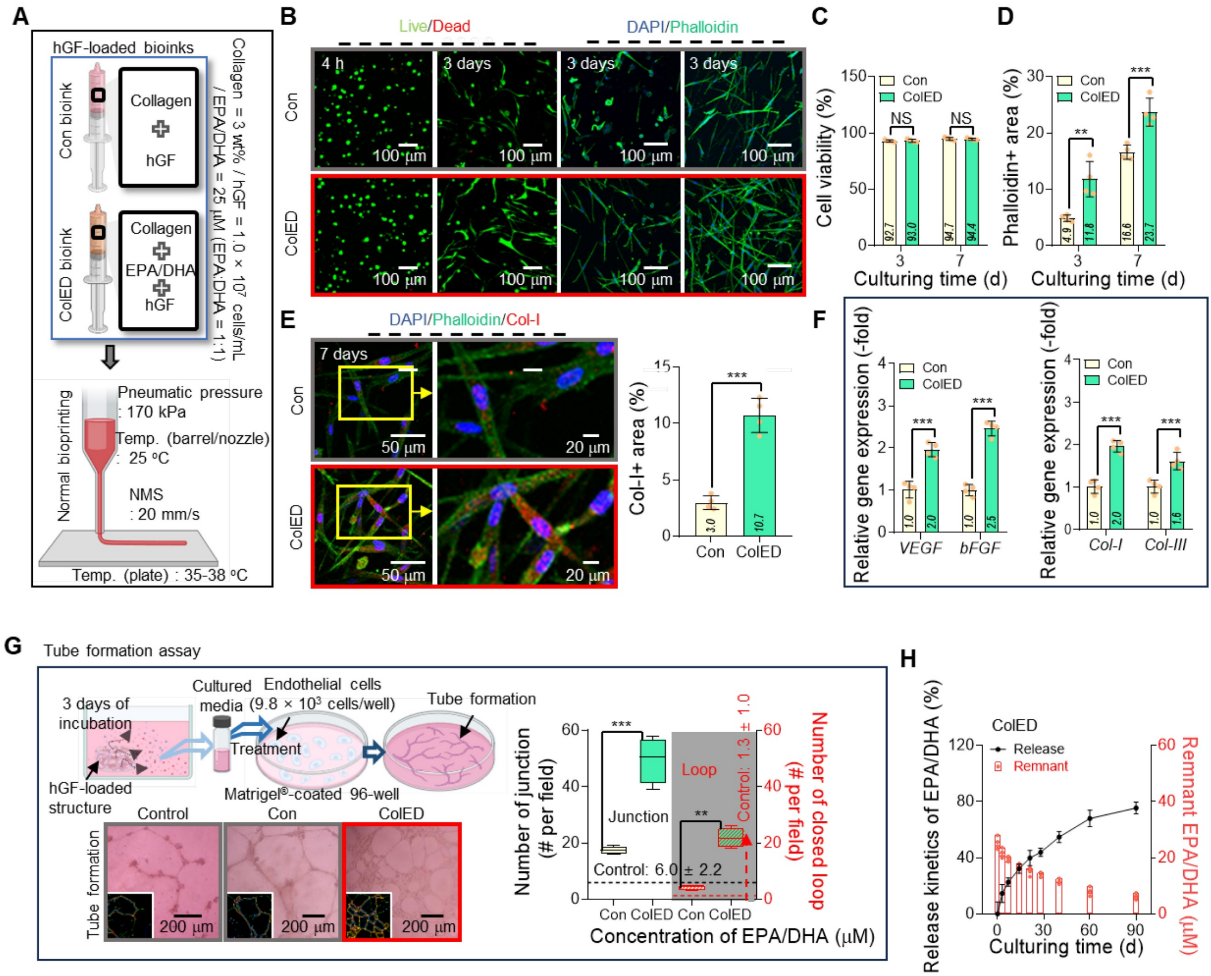
** Gingival tissue regeneration and angiogenic potential of EPA/DHA-loaded cell constructs.** (A) Schematic of the bioprinting process for fabricating control constructs (Con) and EPA/DHA-loaded constructs (ColED) using hGFs (1×10⁷ cells/mL). (B) Live/dead staining images at 4 hours and at 3 d, and DAPI/phalloidin staining at 3 and 7 d, showing high cell viability in both groups and enhanced F-actin formation in the ColED constructs. (C) Quantitative analysis of cell viability and (D) positive phalloidin areas with both constructs (n = 4). (E) Immunofluorescence images of co-staining with DAPI, phalloidin, and collagen type I (Col-I, red) at 7 d, demonstrating increased Col-I expression in ColED constructs (n = 4). (F) Relative gene expression of pro-regenerative markers (*bFGF*, *Col-I*, *Col-III*) and pro-angiogenic marker (*VEGF*) shows significant upregulation in ColED constructs compared to that in Con constructs (n = 4). (G) Tube formation assay, including representative images and quantification of closed loops, reveals a superior angiogenic performance in ColED constructs compared to that in Con constructs (n = 4). (H) Cumulative release profile of EPA/DHA from ColED constructs over 90 d indicates a gradual and sustained release without an initial burst (n = 4). All data are mean ± SD (NS = no significance, **p < 0.01, ***p < 0.001, analyzed using Student's t-test).

To validate the biological effectiveness of the EPA/DHA concentration (25 μM), fatty acids were mixed with hGFs (1 × 10^7^ cells/mL)/Col bioink and used to fabricate cell constructs (ColED) through normal bioprinting. The fabricated ColED constructs were compared with a control cell construct (Con) prepared using the same density of hGFs and pure collagen bioink without EPA/DHA (Figure 4A).

Figure 4B shows the live/dead cells at 4 h and 3 d and the DAPI/phalloidin images at 3 and 7 d for the Con and ColED constructs; the results demonstrated that both cell constructs exhibited high cell viability (Figure 4C); however, F-actin formation in ColED was highly enhanced compared to that in the fatty acid-free group (Figure 4D). Furthermore, the EPA/DHA-containing constructs showed enhanced collagen type I (Col-I) expression, as shown in the DAPI/phalloidin/Col-I (red) images at 7 d (Figure 4E). Gene expression analyses further supported these observations, as the levels of pro-regenerative and pro-angiogenic genes, including *bFGF*, *VEGF*, *Col-I*, and *Col-III*, were significantly elevated in the EPA/DHA-contained constructs compared with those in the Con (Figure 4F). The results demonstrated the role of EPA/DHA in promoting cellular activity and matrix production, both of which are critical for gingival tissue regeneration.

To further investigate the angiogenic potential of EPA/DHA, a tube formation assay was conducted using the cell-constructs (Con and ColED) (Figure 4G). As anticipated, the constructs prepared with the EPA/DHA-containing bioink demonstrated superior angiogenic performance, as evidenced by the higher number of junctions and closed loops compared to those in the Con. These results reinforce the hypothesis that EPA/DHA can enhance endothelial cell activity and support the formation of vascular-like structures through their proangiogenic properties.

Further, to investigate the release behavior of EPA/DHA from the bioprinted ColED, the cumulative release profile of EPA/DHA was analyzed over a 90-d period (Figure 4H). The results revealed a gradual and sustained release of EPA/DHA, with no significant initial burst. The absence of an initial burst release may be important, as it minimizes the risk of cytotoxicity associated with a sudden high concentration of fatty acids while maintaining consistent bioavailability 36,50. Consistent with the release behavior, the reduction in the EPA/DHA droplets also occurred gradually without a severe initial loss, as evaluated by observing the changes in the Nile Red-positive area over the culture period (0-14 d) (Figure S3). We suggest that this phenomenon is due to the physical encapsulation of EPA/DHA droplets by collagen molecules, leading to their gradual and sustained release kinetics 47,48. This prolonged release aligns well with the time frame required for gingival tissue organization and vascular network formation by ensuring prolonged activation of signaling pathways, such as the VEGF and FGF pathways, ensuring that the cell-constructs can provide continuous support for regenerative activities.

### Effects of comb-assisted bioprinting on cell alignment and mechanotransduction

**Figure 5:**
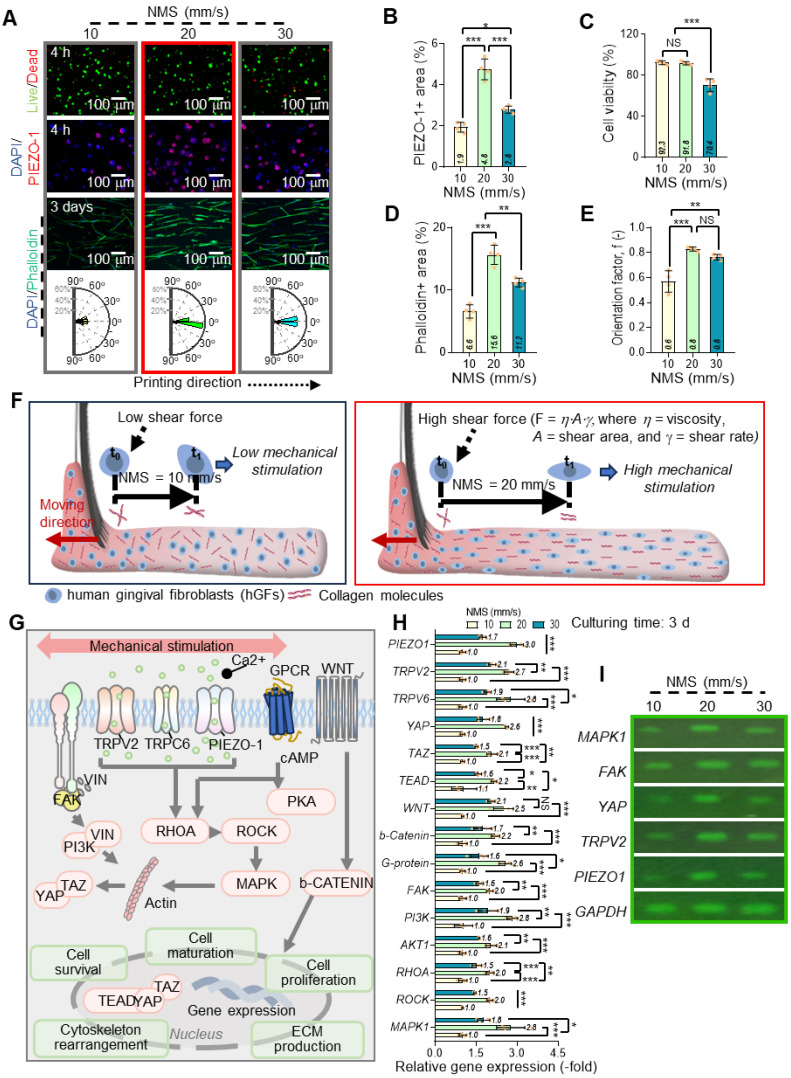
**Effects of NMS on cellular behavior and mechanotransduction in c-bioprinted hGFs.** (A) Live/dead, DAPI/PIEZO-1, and DAPI/F-actin staining images, with rose diagrams showing enhanced alignment at higher NMS (10-30 mm/s). (B) PIEZO-1 expression peaks at intermediate NMS, decreasing at 30 mm/s because of excessive shear forces (n = 4). (C) Cell viability declines at 30 mm/s (n = 4). (D) F-actin formation increases with intermediate NMS, declining at 30 mm/s (n = 4). (E) Orientation factors show greater cell alignment at higher shear forces (n = 4). (F) Schematic of c-bioprinting showing shear stress promoting hGF and collagen fibril alignment with increasing NMS. (G) Schematic of mechanotransduction pathways (PIEZO-1, TRPV2, TRPC6, RHOA, ROCK, PI3K, MAPK, WNT/β-catenin, YAP/TAZ). (H) Gene expression (n = 4) and (I) agarose gel electrophoresis confirming increased mechanotransduction at intermediate NMS and suppression at 30 mm/s. All data are mean ± SD (NS = no significance, *p < 0.05, **p < 0.01, ***p < 0.001, analyzed using ANOVA with Tukey's HSD post-hoc test).

In the c-bioprinting process, one of the key variables under the printing conditions is the nozzle moving speed (NMS), which plays a substantial role in influencing the cellular behavior of hGFs. Figures 5A shows live (green)/dead (red), DAPI (blue)/PIEZO-1 (green), and DAPI (blue)/F-actin (red) staining images taken after 4 h and 3 d of cell culture for hGFs within the ColED cell-constructs fabricated using various NMSs (ranging from 10 to 30 mm/s). A rose diagram showing the frequency of the cell nucleus alignment for each nozzle speed is also shown. As seen in the DAPI/PIEZO-1 images at 4 h, increasing the nozzle moving speed was associated with higher PIEZO-1 expression, suggesting the involvement of mechanosensitive ion channel proteins in response to mechanical forces by facilitating ion flow into cells and triggering intracellular signaling pathways. However, when the NMS reached 30 mm/s, PIEZO-1 expression decreased (Figure 5B), likely because of lower cell viability (Figure 5C), indicating that the excessive shear force caused by high NMS can cause cell damage 51,52.

To directly evaluate the effect of shear stress applied during c-bioprinting on cell viability, the shear stress corresponding to different nozzle moving speeds (NMS) was calculated based on the rheological properties of the ColED bioink. Figure S4A exhibited the results of rheological characterization conducted by varying the shear rate (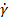) to obtain the shear stress (τ). The power-law model (τ = K
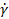
^n^, where K and n are the power-law constants) was utilized to estimate the τ applied during c-bioprinting, and the shear-thinning coefficient, n, was determined from the linear slope of the double-logarithmic plot 53. Using the derived power-law constants (K = 29.4 and n = 0.38), the applied shear stress was estimated based on the calculated shear rate, which was determined by dividing the blade moving speed by the gap distance (h) (Figure S4A). Carefully, the average shear stress (Avg. τ) was assumed by taking the mean of the shear stresses calculated at the regions near (*h_1_*) and far from (*h_2_*) the printing plate to simplify the complex distribution of shear stress generated by the comb accessory (Figure S4B). Notably, when the Avg. τ reached 204 Pa during the c-bioprinting process, a significant decrease in cell viability was detected, indicating potential cell damage under high shear stress conditions (Figure S4C).

Further, we measured F-actin formation and the alignment of cell constructs fabricated using various NMSs. Notably, a significant difference in F-actin formation was observed, indicating trend similar to that of the PIEZO-1 results (Figure 5D). However, higher shear forces (increase in NMS) resulted in a higher cell alignment of the cell-constructs (Figure 5E).

During the c-bioprinting process, the shear force generated by the comb structure facilitates the lateral alignment and mechanical stimulation of collagen fibrils and cells within the bioink. The movement of the bristles was influenced by their moment of inertia, the repulsive force from the substrate, and the external force exerted by the printer 54. As the bristles absorbed the bioink, they left a distinct trace on the working plate. Additionally, the shear force (F = f (η, u, A), where η, u, and A represent shear viscosity, nozzle moving speed, and shearing area, respectively) exerted by the NMS resulted in a thin, uniformly stimulated, cell-laden collagen layer. The interaction between the shear force from the bristles and the friction force from the working plate contributed to the mechanical stimulation of both cells and collagen. Consequently, a higher NMS of > 20 mm/s induced greater shear stress than that at 10 mm/s, leading to enhanced mechanical stimulation of the embedded cells, as depicted in Figure 5F. This further suggests that the collagen molecules and stimulated cells are aligned laterally and parallel to the working substrate due to the shear stress exerted by the comb structure.

To explore the biological effects of shear-induced mechanical stimulation, gene expression analysis was conducted on the bioprinted hGFs, considering several cellular responses. Figure 5G shows the biological responses of hGFs following mechanical stimulation. Mechanical stimulation applied during the fabrication process can be transmitted through the ECM and detected by integrins, vinculin (VIN), and stretch-activated ion channels (SACs), such as TRPV2, TRPV6, and PIEZO-1, found in the cellular membrane 55-60.

To determine the mechanotransduction of hGFs under different NMSs, we measured the expression of mechanotransduction-related genes (*PIEZO-1*, *TRPV2*, and *TRPV6*), *FAK*, and various signaling pathways (including *PI3K*, *AKT1*, *RHOA*, *ROCK*, *MAPK1*, *WNT*, *β-catenin*, *YAP*, *TAZ*, and *TEAD*) after 3 d of cell-culture (Figure 5H). A significant increase in gene expression was observed with increasing NMS; however, at a high NMS (30 mm/s), most mechanotransduction-related genes were suppressed. This phenomenon has been well evaluated in several studies 61,62. According to previous studies, beyond a stimulation threshold, mechanical stimulation may become damaging, causing cellular stress and even suppressing gene expression. Moreover, the biological responsiveness of hGFs was confirmed by agarose gel electrophoresis (Figure 5I).

### *In vitro* results to show regenerative potential

After evaluating the effects of the comb-assisted bioprinting process, various *in vitro* cellular activities were determined with the EPA/DHA-containing collagen bioink (3 wt% collagen and 25 μM EPA/DHA) embedded with hGF (1 × 10^7^ cells/mL), which was fabricated using the comb-assisted bioprinting approach. This method provides mechanical stimuli to cells encapsulated in bioconstructs. The bioconstructs stimulated with the comb-structure (S-ColED) were fabricated under specific conditions (NMS = 20 mm/s, pneumatic pressure in the nozzle = 170 kPa, barrel/nozzle temperature = 25 °C, and plate temperature = 35-38 ℃). To understand the biological impact of the c-bioprinting process, a construct (N-ColED) was prepared using the normal bioprinting method under the same printing conditions.

**Figure 6:**
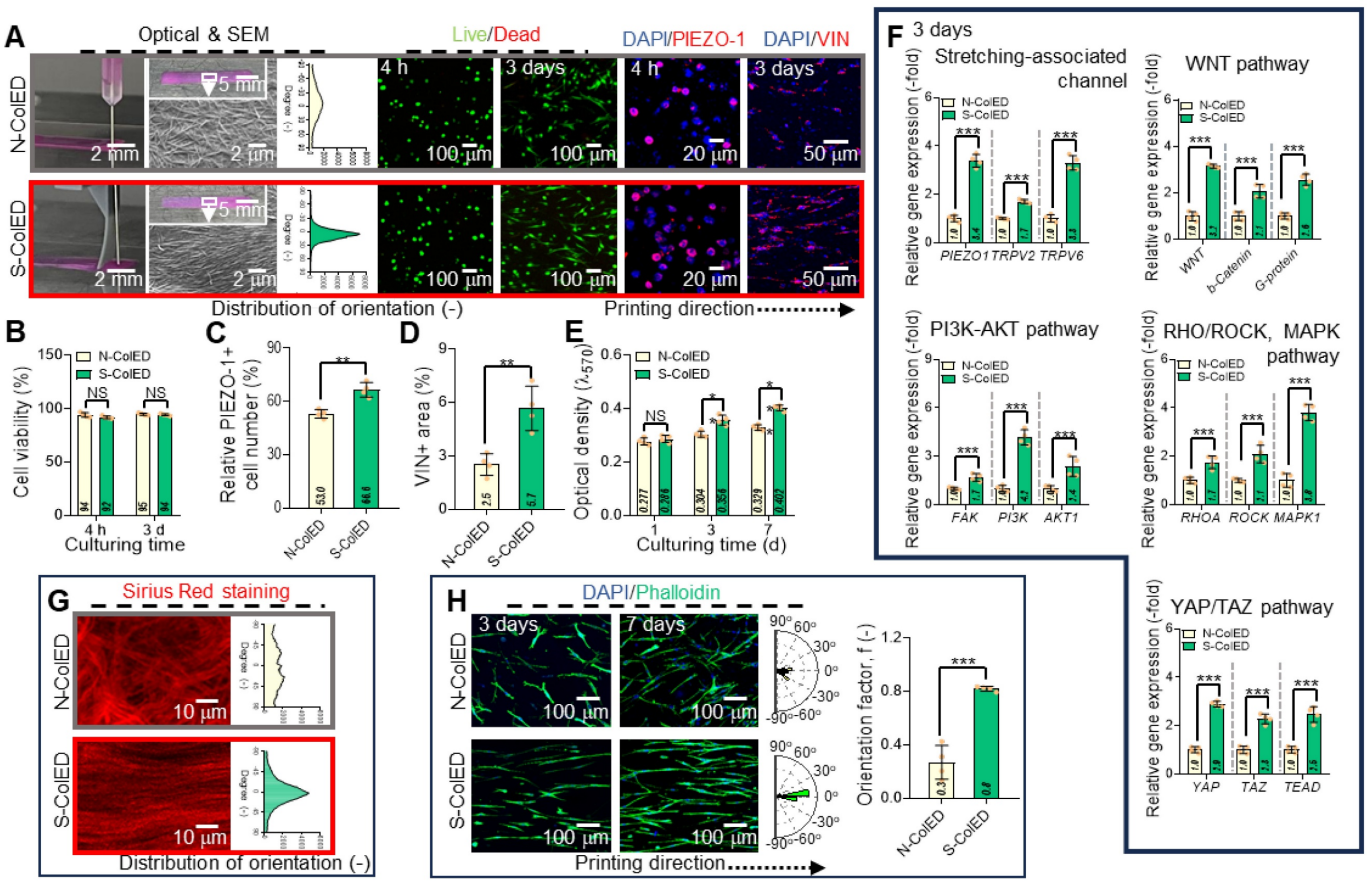
** Evaluation of comb-assisted bioprinting on the cellular activity and alignment of hGFs.** (A) Optical and SEM images of bioprinted constructs using normal (conventional) and comb-assisted methods. Live/dead staining images (4 h and 3 d) show high cell viability (>92%) and enhanced alignment of hGFs and collagen fibrils in comb-assisted constructs. SEM reveals well-organized fibrils (diameter = 241 ± 115 nm; n = 100) in the stimulated group (S-ColED) compared to randomly distributed fibrils (diameter = 235 ± 97 nm; n = 100) in the control (N-ColED). (B) Quantitative analysis of cell viability (n = 4). (C) DAPI/PIEZO-1 immunofluorescence showing enhanced mechanosensing in S-ColED constructs (n = 4). (D) DAPI/VIN (vinculin, red) staining images showing elevated vinculin expression in the S-ColED group (n = 4). (E) Cell proliferation rates significantly higher in comb-assisted constructs (n = 4). (F) Activation of signaling pathways, including PIEZO-1, TRPV2, TRPV6, WNT/β-catenin, G-protein, FAK, PI3K/AKT, RHOA/ROCK, MAPK, and YAP/TAZ (n = 4). (G) Sirius red staining showing collagen fibril alignment in S-ColED constructs. (H) DAPI/phalloidin images (3 and 7 d) show enhanced hGF alignment in comb-assisted constructs (n = 4). All data are mean ± SD (NS = no significance, *p < 0.05, **p < 0.01, ***p < 0.001, analyzed using Student's t-test). The results show that comb-assisted bioprinting improves cell alignment, growth, mechanosensing, and collagen organization.

Figure 6A shows the optical and scanning electron microscopy (SEM) images after *in situ* printing, live/dead (after 4 h and 3 d of culture), DAPI/PIEZO-1, and DAPI/vin (red) staining images for both the N-ColED and S-ColED constructs (20 × 3 × 0.2 mm^3^). In the SEM images, collagen fibrils in the S-ColED group were well-organized (diameter = 241 ± 115 nm), whereas those in the N-ColED group appeared more randomly distributed (diameter = 235 ± 97 nm). Live/dead staining further showed that both the N-ColED and S-ColED bioconstructs had high cell-viability (> 92%) (Figure 6B). In addition, to access the structural stability, we observe the shrinkage of c-bioprinted gingival tissue by incubation in the culture medium.

To assess structural stability, the bioprinted S-ColED constructs were incubated in culture medium. As collagen hydrogels are commonly known to exhibit severe contraction, as reported in previous studies 63-65, the S-ColED showed volume shrinkage of 7.7 ± 1.9% and 14.1 ± 2.8% after 7 and 14 d of culture, respectively. Although the shrinkage remained below 10% during the first 7 d of culture, further studies are planned to address and minimize this contraction in future applications.

Immunofluorescence imaging for DAPI/PIEZO-1 and DAPI/VIN (red) revealed the mechanosensing activity of hGFs after the bioprinting procedure (Figure 6C-D). The results showed a significant increase in PIEZO-1 and VIN expression in the S-ColED group compared with that in the N-ColED group. Furthermore, in the cell growth aspect, the simulated group showed much higher cell proliferation than that in the N-ColED group owing to enhanced cellular microenvironment suitability or various signaling pathways (Figure 6E). To evaluate the signaling pathways induced by mechanical stimulation, mechanosensitive channels (PIEZO-1, TRPV2, TRPV6), WNT/β-catenin including glycoprotein (G-protein), FAK activation, PI3K/AKT, RHOA/ROCK, MAPK, and YAP/TAZ, were measured (Figure 6F). Notably, the initial PIEZO-1 expression was steadily downregulated in cells cultured on both bioconstructs; however, expression levels remained significantly higher in S-ColED compared to N-ColED (Figure S5). We hypothesize that the sustained mechanotransduction observed in S-ColED is attributed to the presence of organized collagen fibrils 66,67. As shown in the results, activation of these pathways clearly showed the effect of the c-bioprinting process. The results confirmed that mechanical stimulation from the c-bioprinting process significantly enhanced cell growth, cytoskeletal organization, and ECM production of bioprinted hGFs. Further, we measured the effect of c-bioprinting on hGF alignment in the printing direction. As expected, the collagen fibrils shown in the Sirius red staining images captured after in situ c-bioprinting were significantly aligned with the printing direction compared to those processed by normal printing (Figure 6G). Along with the collagen fibrils, hGFs were aligned in the printing direction, as shown in the DAPI/phalloidin images on days 3 and 7 (Figure 6H). These results suggest that the well-organized collagen fibrils produced by the c-bioprinter guided the directional growth of the bioprinted hGFs, facilitating their alignment.

**Figure 7:**
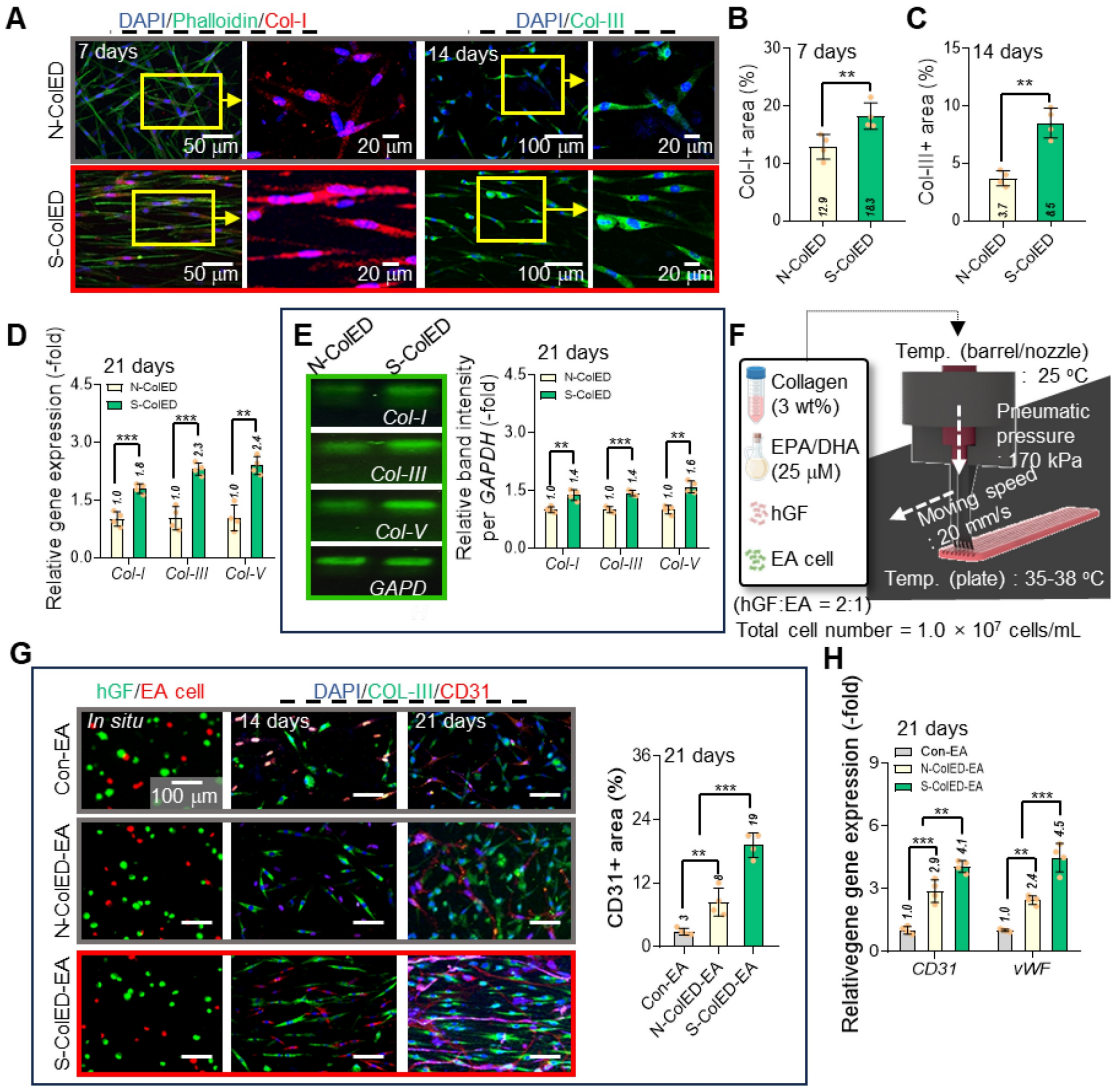
** Enhanced ECM formation and gingival tissue and vessel network development in bioprinted constructs.** (A) Immunostaining images of collagen type-I (Col-I) and collagen type-III (Col-III) in control and comb-assisted constructs. Quantitative analysis showing significantly increased (B) Col-I and (C) Col-III expression in the stimulated group (n = 4). (D) Gene expression and (E) agarose gel electrophoresis results for collagen types (I, III, and V) (n = 4). (F) Schematic of bioink formulation containing endothelial cells and the printing condition of comb-assisted bioprinting. (F) Immunostaining images [hGF (green) and EA (red) using cell-tracker, DAPI/Col-III (green)/CD31 (red)] and positive CD31 area of bioconstructs containing hGF and EA cells, fabricated using the normal (N-ColED-EA) and c-printing process (S-ColED-EA) (n = 4). Normally bioprinted hGF/EA-loaded collagen construct (Con-EA construct) was used as the control. (H)h Gene expression results of *CD31* and *vWF* (n = 4). All data are mean ± SD (**p < 0.01, ***p < 0.001, analyzed using Student's t-test).

To observe proper ECM formation and functional gingival tissue development, Col-I and Col-III (green) were measured in the N-ColED and S-ColED cell constructs (Figure 7A). The S-ColED group exhibited significantly improved Col-I (Figure 7B) and Col-III (Figure 7C) expressions compared to that in the N-ColED group. Gene expression and agarose gel electrophoresis for collagen types (I, III, and V) were also performed (Figure 7D-E), and the expression results matched well with the immunostaining results. Additionally, S-ColED exhibited higher mechanical properties, with a Young's modulus of 211 ± 35 kPa compared to 92 ± 18 kPa for N-ColED after 14 days of culture, indicating more robust ECM formation 68, although these values are still lower than those of native tissue (Figure S6B-C). These results suggest that comb bioprinting can effectively encourage cellular mechanotransduction in bioprinted constructs and even promote a well-organized ECM network, contributing to proper fibrillogenesis and maintaining the functional integrity of the gingival tissue.

To evaluate the efficacy of the c-bioprinting process in generating angiogenesis in the bioconstructs, we fabricated cell-constructs containing two cells (hGFs and endothelial cells (EA)) with a mixture ratio of 2:1, as shown in Figure 7F. As a control, a GF/EA-loaded collagen construct (Con-EA) was bioprinted using the standard process. Based on the immunofluorescence images (hGF (green) and EA (red) images using a cell tracker and DAPI/Col-III/CD31 images), the angiogenesis of the S-ColED-EA group using c-bioprinting was more highly improved than that of the normal bioprinting process (Figure 7G). This result is consistent with the gene expression data for *CD31* and von Willebrand Factor (*vWF*) (Figure 7H). This phenomenon occurs because shear-induced bioprinting can enhance angiogenesis by activating endothelial mechanotransduction, improving cell-matrix interactions, facilitating better endothelial network formation, and eventually upregulating various angiogenic genes 69-71. Moreover, the bioconstructs containing EPA/DHA (N-ColED-EA and S-ColED-EA) exhibited enhanced expression of angiogenesis-related markers compared to those in the Con-EA group, indicating that incorporation of EPA/DHA can promote the vascularization of bioprinted gingival tissue.

### *In vivo* evaluation of comb-printed gingival constructs

**Figure 8:**
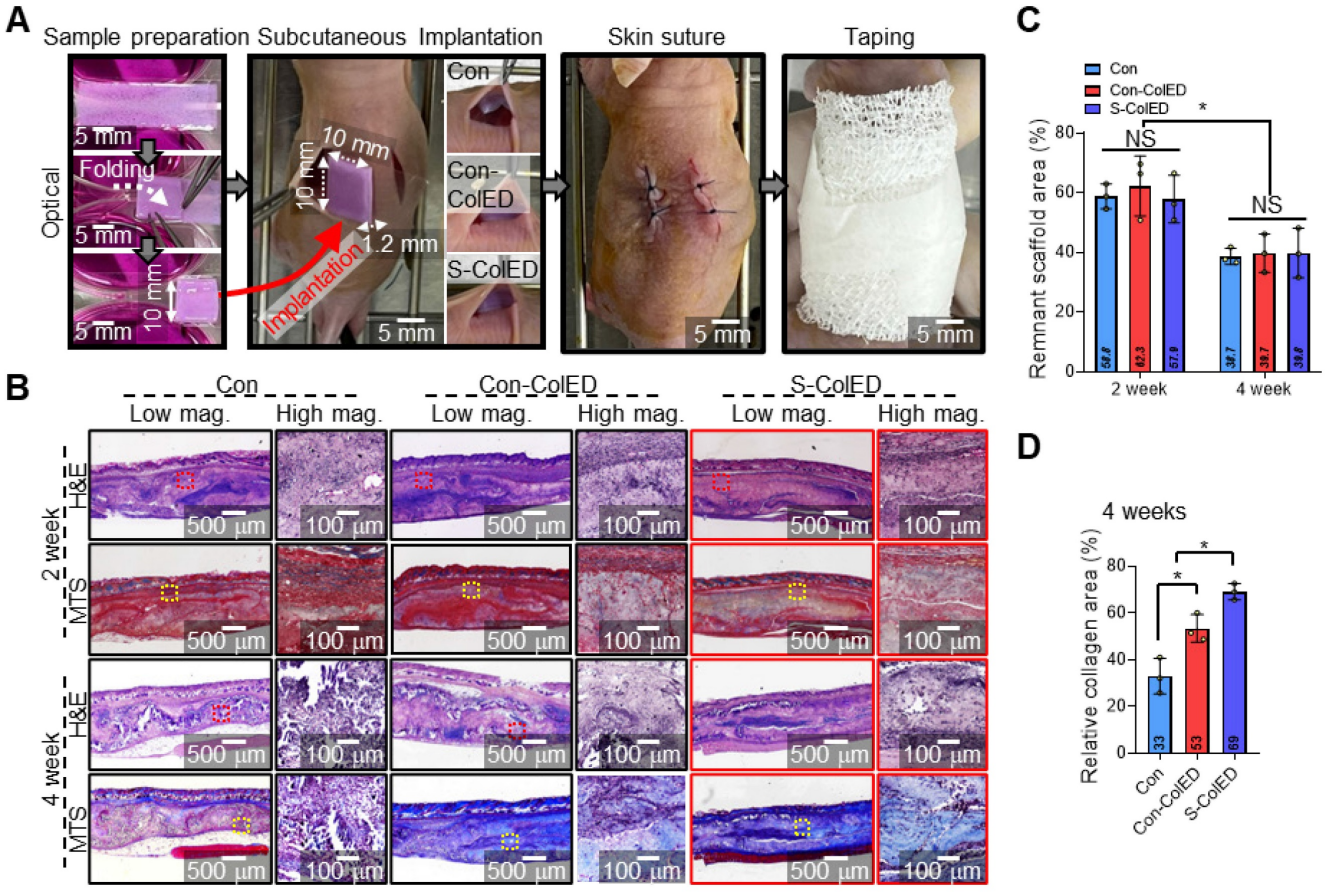
** Histological analysis of the bioprinted gingival construct in subcutaneous region of mice.** (A) Schematic of sample preparation and subcutaneous implantation. (B) Hematoxylin and eosin (H&E) staining and Masson's trichrome staining (MTS) of the implantation site, show tissue morphology and collagen deposition. (C) Quantitative assessment of the remaining scaffold area at 2 and 4 weeks post-implantation (n = 3). (D) Quantified relative collagen area at 4 weeks post-implantation (n = 3). All data are mean ± SD (NS = no significance, *p < 0.05, **p < 0.01, analyzed using ANOVA with Tukey's HSD post-hoc test).

To assess vessel infiltration and gingival tissue formation, a comparative study was conducted using bioprinted structures containing hGFs (Con, Con-ColED, and S-ColED), which were implanted subcutaneously into the dorsal area of nude mice as shown in Figure 8A. In the present study, evaluation within a subcutaneous environment as a preliminary step before conducting regeneration studies using a gingival defect animal model, and nude mice, frequently used for subcutaneous implantation, were employed to avoid immune rejection of human cells 72,73. The hGFs-loaded bioconstructs (10 × 40 × 0.3 mm^3^) were folded manually into a volumetric structure (10 × 10 × 1.2 mm^3^) before implantation. Throughout the experimental period, no signs of toxicity or adverse effects were observed, and the animals remained healthy. The mice were sacrificed to evaluate vascularization and gingival tissue formation, and tissues were harvested for histological and immunochemical staining analyses after 2- and 4-weeks post-implantation. Figure 8B shows the histological images of Hematoxylin and Eosin (H&E) staining and Masson trichrome staining (MTS) of Con, Con-ColED, and S-ColED groups. The collagen-based structures were gradually degraded over experimental periods in all three groups, with no significant differences in the remaining bioconstruct area, which was less than 40% after 4 weeks of implantation (Figure 8C). Furthermore, collagen area measurements using MT staining images of the implant site revealed 2.1-fold and 1.3-fold increases in collagen area in mice that received S-ColED, compared to the Con and Con-ColED bioconstruct groups, respectively (Figure 8D). These findings suggest that the hGFs in S-ColED significantly contributed to collagen deposition, thereby enhancing the gingival tissue formation 74.

**Figure 9:**
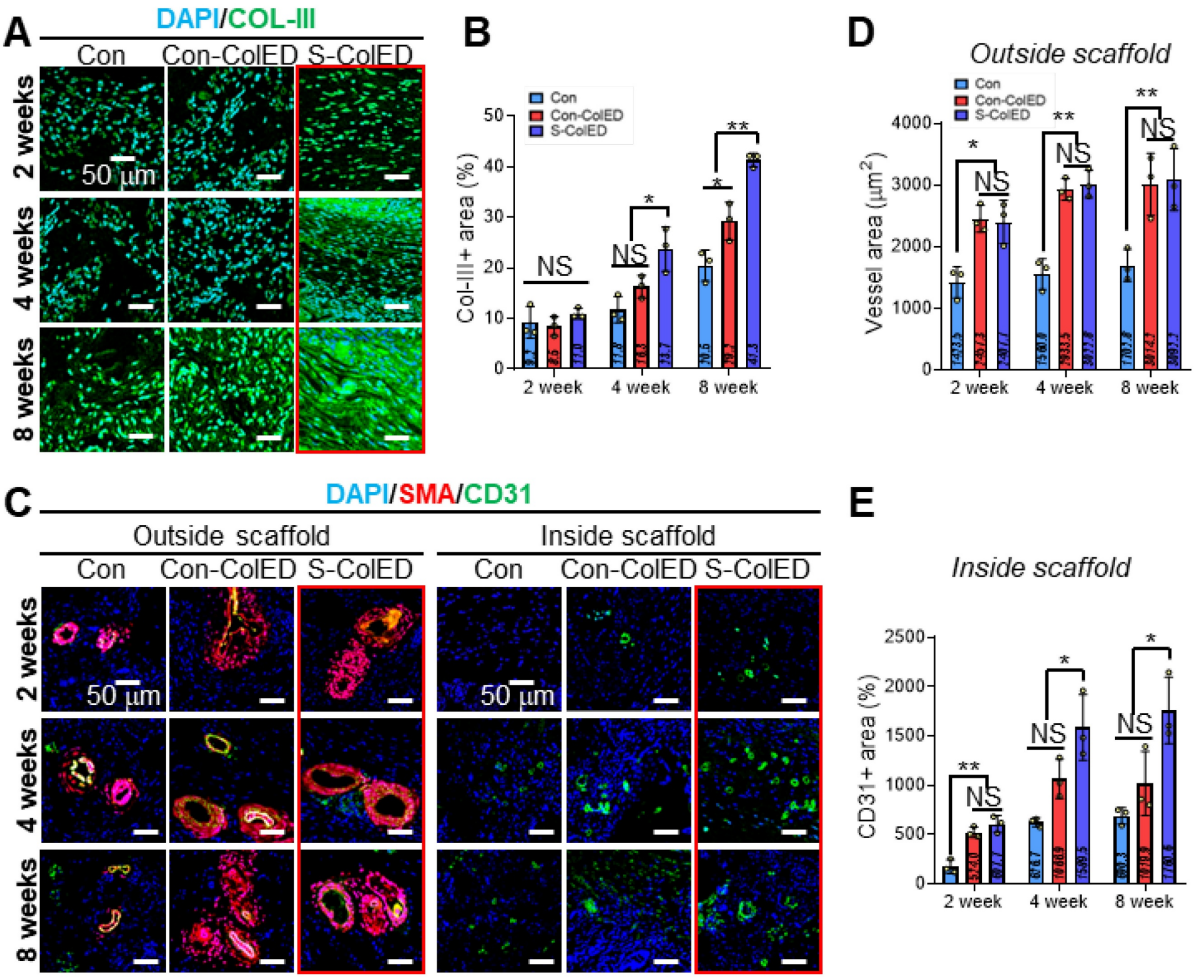
** Immunochemical analysis of the bioprinted gingival construct in subcutaneous region of mice.** (A) Immunohistochemical staining of DAPI (blue) and collagen III (Col-III, green) to assess tissue formation. (B) Quantification of the Col-III area at 2, 4, and 8 weeks post-implantation (n = 3). (C) Immunohistochemical staining of DAPI (blue), smooth muscle actin (α-SMA, red), and CD31 (green) to visualize the inner and outer regions of the implanted scaffolds. (D) Measurement of vessel area in the outer region (n = 3), and (E) quantification of CD31-positive area in the inner region at 2, 4, and 8 weeks after implantation (n = 3). All data are mean ± SD (NS = no significance, *p < 0.05, **p < 0.01, ***p < 0.001, analyzed using ANOVA with Tukey's HSD post-hoc test).

To assess vascularization and gingival tissue formation, immunochemical staining was performed using Col-III (red) and smooth muscle alpha-actinin (α-SMA, red)/CD31 (green) antibodies, as these markers are expressed in hGFs and vessels, respectively (Figure 9) 74,75. Figure 9A demonstrates the DAPI/Col-III images of hGFs within the bioprinted Con, Con-ColED, and S-ColED at 2, 4, and 8 weeks post-implantation. While similar levels of Col-III expression were observed across all groups at 2 weeks, significantly higher Col-III deposition was detected in the EPA/DHA-assisted groups compared to the Con structure as validated by Col-III-positive area at 4 and 8 weeks (Figure 9B). Notably, hGFs received initial mechanical stimulation through the comb-printing process and exhibited the highest Col-III deposition in the subcutaneous environment.

In general, blood vessel infiltration/formation within the implanted scaffold is one of the key factors, as it facilitates effective graft-host interactions and supports the long-term survival of delivered cells 75. Figure 9C shows the DAPI/α-SMA/CD31 of the outer and inner regions of the implanted gingival tissues after 2, 4, and 8 weeks. As expected, the EPA/DHA-assisted hGF-constructs exhibited enhanced vascular formation and infiltration compared to the Con group. Relatively larger peripheral blood vessels positive for α-SMA and CD31 staining were observed surrounding both Con-ColED (3014 ± 504 μm^2^ at 8 weeks) and S-ColED (3092 ± 501 μm^2^ at 8 weeks) groups compared to Con structure (1703 ± 259 μm^2^ at 8 weeks) (Figure 9D). In particular, the infiltration of capillaries positive for CD31 staining but negative for α-SMA into the interior of the constructs was significantly enhanced in the EPA/DHA-containing groups at 8 weeks post-implantation, with a greater level observed in the S-ConED group, as confirmed by quantification of the CD31+ area (Con: 690 ± 85 μm^2^, Con-ColED: 1020 ± 326 μm^2^, and S-ColED: 1761 ± 337 μm^2^) (Figure 9E). These results, consistent with the results of *in vitro* biological responses including angiogenic factor expression, tube formation assay, and collagen expression, suggest that the introduction of EPA/DHA, which supports angiogenesis, along with the initial mechanical stimulation provided by the comb-printing method, can regulate collagen deposition by hGFs and promote vessel formation within the *in vivo* subcutaneous environment. Although the subcutaneous implantation test demonstrated the angiogenic potential of the S-ColED constructs, there remains a gap in vessel size compared to native gingival tissue 19, highlighting the need for further validation of vascular formation within a clinically relevant gingival tissue model.

Through both *in vitro* and subcutaneous *in vivo* studies, the potential application of the c-bioprinted S-ColED constructs in periodontal plastic surgery and tissue engineering has been further substantiated. This approach holds promise not only for promoting gingival tissue formation but also for inducing vascularization to restore damaged gingival tissues, thereby addressing needs in periodontal and oral tissue engineering. While the subcutaneous implantation model allowed for the evaluation of regenerative potential, its limitations in replicating the dynamic oral environment highlight the necessity for future validation using surgical gingival defect models to confirm the functional regenerative capacity of the constructs under clinically relevant conditions. In addition, direct comparisons with bioconstructs obtained using existing bioinks are planned to further substantiate the advantages of the S-ColED.

## Conclusion

This study demonstrates the potential of using 3D comb-assisted bioprinting technology combined with biochemical and biophysical strategies for gingival tissue regeneration. Using a collagen-based bioink incorporating hGFs and omega-3 fatty acids, the engineered bioconstructs successfully enhanced essential regenerative processes, including cell growth, collagen production, and angiogenesis. The incorporation of a comb-attached bioprinting system further enabled the precise alignment of hGFs and mechanotransduction, thereby activating several key signaling pathways. *In vitro* analyses confirmed the ability of the bioconstruct to promote gene expression associated with collagen production and tissue formation, whereas *in vivo* transplantation revealed the formation of blood vessel-like structures, underlining its regenerative potential. These results highlight the importance of integrating bioprinting techniques with specific bioinks and mechanical cues, offering a potential pathway for developing effective strategies in gingival tissue engineering.

## Materials and Methods

### Cell culture and bioink formulation

Human gingival fibroblasts (hGFs; ATCC^®^, USA) and EA.hy926 endothelial cells (EA cells; ATCC^®^, USA) were cultured in Dulbecco's modified Eagle medium-high glucose (DMEM-hg; Sigma-Aldrich, USA) containing 10% fetal bovine serum (FBS; Biowest, France) and 1% penicillin/streptomycin (PS; Gibco, USA) before formulating the cell-laden bioinks. The cells were cultured at 37 °C under a 5% CO_2_ environment, with the media replaced every 2 d.

To print the gingival construct, a collagen-based bioink supplemented with a mixture of eicosapentaenoic acid (EPA; Sigma-Aldrich, USA) and docosahexaenoic acid (DHA; Sigma-Aldrich, USA) was prepared. Briefly, a collagen type-I sponge (MSBio, South Korea) derived from porcine skin was dissolved in distilled water (DW) and neutralized by mixing with a 10× enriched DMEM solution (Gibco, USA) at a ratio of 1:1 51,76. Then, a mixture of EPA/DHA (1:1) and hGFs (1.0 × 10^7^ cells/mL) was mixed with a collagen (3 wt%) hydrogel. To evaluate the effects of EPA/DHA on cellular activity, their concentrations were 10, 25, 50, and 100 μM. As a control, hGF (1 × 10^7^ cells/mL)-loaded collagen (3 wt%) bioink without EPA/DHA was used to determine the *in vitro* cellular activity of the collagen bioink supplemented with EPA/DHA. Further, a mixture of hGFs/EA cell (total cell density = 1.0 × 10^7^ cells/mL; hGF:EA cells = 2:1 77)-loaded collagen (3 wt%)-EPA/DHA (25 μM) bioink was prepared to print the pre-vascularized gingival tissue.

To further verify the biological feasibility of collagen-based bioink, a porcine gingival tissue-derived decellularized extracellular matrix (GdECM) was prepared using slightly modified methods based on previously reported protocols 51,78. To prepare GdECM, porcine gingival tissue was isolated and cut into small discs (approximately 5 × 5 × 1 mm^3^). The decellularization procedure involved treatment with 1 wt% sodium dodecyl sulfate (SDS; Sigma-Aldrich, USA) solution for 5 d until the color of the tissue turned completely white, followed by treatment with 0.5% Triton X-100 (Sigma-Aldrich, USA) for 1 d and with isopropanol (Sigma-Aldrich, USA) for 12 h to remove lipids. DW and Dulbecco's phosphate buffered saline (DPBS) were used to rinse the sample and hypotonic/hypertonic treatments were then performed for 2 h each, with three changes per day, over a period of 3 d. The tissues were then lyophilized at -80 °C for 2 d. To solubilize the GdECM, the freeze-dried tissues were digested in 0.5M acetic acid solution (Sigma-Aldrich, USA) containing pepsin (1 mg/ml) under continuous stirring for at least 48 h, precipitated by adding sodium chloride (Sigma-Aldrich, USA) under continuous stirring, centrifuged (3000 rpm) for 15 min to obtain a large protein pellet, dialyzed using a dialysis tubing (molecular weight cut-off = 3,500 kDa; Spectra/Por^TM^, USA) for 2 d at 4 °C, and freeze-dried. The tissues were washed thrice with DPBS and DW between chemical treatments.

After preparing the GdECM, the soluble collagen, glycosaminoglycans, and elastin contents in the tissues were quantitatively determined using Sircol^TM^ Soluble Collagen, Blycan^TM^ Sulfated Glycosaminoglycan, and Fastin^TM^ Elastin (Biocolor Life Sciences Assays, UK) assays, according to the respective manufacturer's protocols 79. The prepared GdECM sponge was dissolved in DW, neutralized by mixing with 10× enriched DMEM solution at a ratio of 1:1, and mixed with hGFs (1 × 10^7^ cells/mL) to obtain a final GdECM concentration of 3 wt%. All values are expressed as the mean ± standard deviation (SD) (n = 3).

### Bioprinting of aligned gingival tissues using the comb-casting process

In this study, a comb-assisted bioprinting process that can activate cellular mechanotransduction through mechanical stimulation was performed to obtain a gingival construct containing aligned hGFs. A comb attachment (bristle gap = 400 μm, bristle length = 1 mm) for the bioprinting nozzle was printed using a 3D printer (Cubicon Single Plus, Cubicon Inc., South Korea) supplemented with an acrylonitrile butadiene styrene filament (ABS_A100, Cubicon Inc.) to support the modified bioprinting method. Computer-aided design (CAD) software (Shapr3D; Sharp3D.Zrt, Hungary) and the official software (Cubicreator4 V4.2.4, Cubicon Inc., South Korea) were used to generate standard tessellation language files. Before application, the printed comb accessory was sterilized several times using 70% ethanol and DW in a UV environment.

To print the aligned hGF-loaded construct, a 3D bioprinting system (DTR3-221T-EX; DASA Robot, South Korea) assisted with a pneumatic pressure dispenser (AD-3000C; Ugin-tech, South Korea) and a comb-attached microscale nozzle (inner diameter = 200 μm; NanoNC, South Korea) was performed under controlled barrel/nozzle (25 °C) and fabricating plate (35-38 °C) temperatures and pneumatic pressures (170 kPa). Various nozzle moving speeds were tested to select the appropriate fabrication parameters for stimulating and aligning hGFs. As a control, a normal bioprinting process without the comb accessory was performed under the same bioprinting conditions to evaluate the biological activities of comb-assisted bioconstructs.

### Evaluation of bioink and gingival construct

To observe the morphologies of bioprinted constructs, native gingival tissue, and the prepared GdECM, an optical microscope (BX FM-32; Olympus, Japan) supplemented with a single-lens reflex digital camera (Canon, Japan) and a scanning electron microscope (SEM) (SNE-3000M; SEC Inc., South Korea) were used.

After conducting comb-assisted bioprinting, the aligned collagen fibrils were visualized by Sirius red (SR) staining. The collagen-based samples were fixed and permeabilized using neutral buffered formalin (NBF) (10% in DPBS; Sigma-Aldrich, USA) for 1 h and Triton X-100 (2%; Sigma-Aldrich, USA) for 20 min at 37°C, respectively. The treated specimens were then incubated in the SR solution for 40 min at 37°C. The stained collagen fibers were visualized using a confocal microscope (LSM 700; Carl Zeiss, Germany) and their orientation trends were assessed using ImageJ software (National Institutes of Health, USA). All values are presented as the mean ± SD (n = 4; three randomly captured images of four different specimens from each group).

Rheological properties (storage modulus, G' and shear viscosity, η) of the collagen-based bioinks containing diverse concentrations of EPA/DHA (10, 25, 50, and 100 μM) were evaluated using a rotational rheometer (Bohlin Gemini HR Nano; Malvern Instruments, UK) supplemented with a cone-and-plate geometry (cone angle: 4°, diameter: 40 mm, and gap: 150 μm) with shear rate (strain: 1%, temperature: 25 °C, and shear rate range: 0.1-100 s^-1^), frequency (strain: 1%, temperature: 25 °C, and frequency range: 0.1-10 Hz), and temperature (strain: 1%, frequency: 1 Hz, temperature range: 5-45 °C, and ramping rate: 1 °C/min) sweeps. All values are expressed as the mean ± SD (n = 3).

The release kinetics of the ColED bioink were evaluated by estimating the remnant EPA/DHA concentrations using a Free Fatty Acid Quantification Assay Kit (Abcam, USA) according to the manufacturer's protocols. A microplate reader (Epoch; BioTek, South Korea) was used to measure the EPA/DHA values at 570 nm. Known free fatty acid standards were used to calculate the EPA/DHA concentrations. The value for the collagen bioink without EPA/DHA was estimated using an assay kit to normalize the EPA/DHA content. All values are exhibited as the mean ± SD (n = 4).

To visualize the O-W emulsion, EPA/DHA components were stained with Nile red solution (Sigma-Aldrich, USA) based on the manufacture's protocol. Briefly, EPA/DHA-assisted bioink was incubated in the Nile red solution (0.5 μg/mL in DPBS) for 30 min h at 37 ^o^C. The stained droplets were visualized using the Carl Zeiss confocal microscope and Nile red+ area was quantified using the ImageJ software. All values are presented as the mean ± SD (n = 4; three randomly captured images of four different specimens from each group).

### Assessment of *in vitro* cellular responses

After formulating the bioinks and conducting the bioprinting processes, the cells were stained using live (green)/dead (red) solution, consisting of 0.15 mM calcein AM (Invitrogen, USA) and 2 mM ethidium homodimer-1 (Invitrogen, USA) dissolved in DPBS, to observe cell viability. The stained live and dead cells were visualized using an LSM 700 microscope, and the captured images were used to quantify cell viability by counting the number of green- and red-colored cells using ImageJ software. All values are presented as the mean ± SD (n = 4; three randomly captured images of four different samples from each group).

To observe cellular orientation and actin filament (F-actin) formation, cell nuclei and F-actin were stained with 4′,6-diamidino-2-phenylindole (DAPI; blue) (1:100 in DPBS; Invitrogen, USA) and fluorescein phalloidin (green) (1:100 in DPBS; Invitrogen, USA) staining solution. The cell-loaded samples were fixed and permeabilized using 10% NBF for 1 h and 0.5% Triton X-100 for 20 min at 37 °C, respectively, before staining. In addition, cells were stained with 2′,7′-Dichlorofluorescein diacetate (DCF-DA) (Sigma-Aldrich, USA) to observe the reactive oxygen species (ROS) deposition based on the manufacture's protocol. A Carl Zeiss microscope was used to visualize the cells, followed by quantitative evaluation of the phalloidin+ area, cellular alignment trend, and orientation factor using ImageJ software. All values are presented as the mean ± SD (n = 4; four randomly captured images of three different samples for each group).

In this study, the proliferation of the bioprinted cells was assessed by observing cellular metabolism using the Cell Proliferation Kit I (Boehringer, Germany). Following incubation of the bioconstructs with a pre-warmed 3-(4,5-dimethylthiazol-2-yl)-2,5-diphenyltetrazolium bromide (MTT) working solution (0.5 mg/mL in media) for 4 h at 37 °C to form purple formazan crystals, stop solution containing dimethyl sulfoxide (DMSO; Sigma-Aldrich, USA) was treated to dissolve the purple crystals. An Epoch microplate reader was used to measure the optical density of the purple medium at 570 nm. All values are expressed as mean ± SD (n = 4).

To evaluate the angiogenic ability of EPA/DHA-assisted bioinks, the tube formation assay was conducted by seeding EA Cells onto the Matrigel® (Corning, USA)-coated ninety-six-well culture plates. The seeded EA cells (9.8 × 10^3^ cells/well) were cultured with the conditioned media of bioinks supplemented with different EPA/DHA concentrations (10, 25, 50, and 100 μM), which were collected at 3 d of incubation with 1 mL serum-free DMEM-hg at 37 °C in a 5% CO₂ environment after culturing with growth media for 3 d. Tube formation was observed under the optical microscope after 1 d of culture using the conditioned media and pure serum-free DMEM-hg at 37 °C in a 5% CO₂ environment. ImageJ software supplemented with the Angiogenesis Analyzer Plugin was used to quantitatively estimate the number of junctions and closed loop formations 80,81. All values are presented as means ± SD (n = 4; three randomly captured images of four different samples from each group).

To observe the distribution of hGFs and EA cells, the cells were pre-stained with CellTracker^TM^ (Molecular Probes, USA) solution according to the manufacturer's protocol 82. Briefly, the pre-cultured hGFs and EA cells were incubated with the -warmed staining solution (37 °C) consisting of CellTracker™ Green CMFDA (hGFs) and Red CMTPX (EA cells) dyes under a 5% CO_2_ environment at 37 °C for 30 min. Confocal microscopy was used to visualize the distribution of green- and red-stained cells.

The mechanical properties of the bioprinted gingival tissues (5 × 14 × 0.4 mm^3^; at 14 d) and porcine-derived native gingival tissue (5 × 14 × 0.4 mm^3^) were assessed using a universal testing machine (SurTA; Chemilab, South Korea) under tensile mode at a stretching rate of 0.1 mm/s in the wet state. Young's moduli were estimated from the linear regions of the plotted stress-strain (S-S) curves. All data are presented as means ± SD (n = 4).

### Enzyme-linked immunosorbent assay (ELISA)

The vascular endothelial growth factor (VEGF) secretion ability of the EPA/DHA-assisted bioinks was assessed using a human VEGF ELISA kit (Invitrogen, USA) after 3 d of culture, following the manufacturer's protocols 83. After incubation of bioinks containing various concentrations of EPA/DHA (10, 25, 50, and 100 μM) with 1 mL serum-free DMEM-hg for 1 d at 37 °C in a CO_2_ environment, VEGF levels in the conditioned media were measured using ELISA plates and a BioTek microplate reader at 450 nm. Known standards were used to calculate the concentration of secreted VEGF. All values are expressed as mean ± SD (n = 4).

### Immunofluorescence imaging analysis

Immunofluorescence imaging was performed on cell-laden samples, native gingival tissue, and GdECM. The specimens were treated with 0% NBF (for 1 h), 2% Triton X-100 (for 2 h), and 2% bovine serum albumin (BSA; Sigma-Aldrich, USA) (for 2 h) at 37 °C for fixing, permeabilizing, and blocking the samples, respectively. The prepared samples were then incubated overnight at 4 °C with mouse anti-Collagen type-I (Col-I) (5 μg/mL in DPBS; Invitrogen, USA), rabbit anti-Col-III (5 μg/mL in DPBS; Invitrogen, USA), mouse anti-PIEZO-1 (5 μg/mL in DPBS; Invitrogen, USA), mouse anti-vinculin (VIN) (5 μg/mL in DPBS; Invitrogen, USA), mouse anti-CD31 (5 μg/mL in DPBS; Invitrogen, USA), mouse anti-fibronectin (5 μg/mL in DPBS; Invitrogen, USA), mouse anti-elastin (5 μg/mL in DPBS; Invitrogen, USA), and mouse anti-laminin (5 μg/mL in DPBS; Invitrogen, USA) primary antibodies, and stained for 1 h at 37 °C with anti-mouse secondary antibodies conjugated with Alexa Fluor 594 and anti-rabbit secondary antibodies conjugated with Alexa Fluor 488 (1:50 in DPBS; Invitrogen, USA). The nuclei were counterstained using 5 μM DAPI dissolved in DPBS. Stained samples were visualized using a confocal microscope. The captured images were used to quantify the Col-I+, Col-III+, PIEZO-1+, and VIN+ areas using ImageJ software (n = 4; three randomly captured images of four different samples from each group).

### Polymerase chain reaction (PCR) analysis

To evaluate the gene expression from bioprinted gingival tissues, quantitative reverse transcription PCR (qRT-PCR) and DNA electrophoresis were performed. For qRT-PCR, data analysis was conducted based on the Livak method 84-86. Briefly, total RNA was isolated using TRIzol® Reagent (ThermoFisher Scientific, USA), and cDNA was synthesized from RNase-/DNase-free RNA using reverse transcription with the ReverTraAce qPCR RT Master Mix (Toyobo Co., Ltd., Japan). Then, qRT-PCR analysis was conducted using the synthesized cDNA and Thunderbird® SYBER® qPCR mix (Toyobo Co., Ltd., Japan) on the StepOnePlus PCR system (Applied Biosystems, USA). The analysis was completed, and threshold cycle (CT) values were measured after 40 cycles. Glyceraldehyde 3-phosphate dehydrogenase (*GAPDH*) and beta-actin (*ACTB*) were used as housekeeping genes to normalize other CT values. After 30 cycles on the StepOnePlus PCR system, the PCR products were used for DNA electrophoresis. The PCR products were stained with Loading STAR (Dyne Bio, South Korea) to observe the bands, and separated on an agarose gel (1.2%). The intensity of the captured band images was quantitatively estimated using ImageJ software, and the intensity of the *GAPDH* band in each group was used as a control to normalize the values. The primers used are listed in Table 1. All values are presented as mean ± SD (n = 4).

### Animal housing and care

For this *in vivo* animal study, athymic nude mice were housed in the Hallym University Animal Care Facility. All experimental procedures were conducted under a protocol approved by the Institutional Animal Care and Use Committee (IACUC) of Hallym University (Approval No. HMC 2024-2-1209-43). The mice were placed in isolated cages according to their experimental groups. The facility's temperature (22 ± 1 °C) and air supply were controlled and monitored. Animals had free access to food and water, and cage bedding was changed every 2-3 days. Lighting conditions were regulated with an automatic timer, maintaining a 12-hour light/dark cycle (12 h : 12 h).

### *In vivo* surgical procedures for the subcutaneous mouse model

The Con-ColED and S-ColED bioconstructs (10 × 40 × 0.3 mm³) were folded to create three-dimensional volumetric structures (10 × 10 × 1.2 mm³), which were then implanted into dorsal subcutaneous pockets of athymic nude mice (Orient Science Co.). As a control, the Con structure was folded using the same process and implanted.

Each mouse was anesthetized with 2.5% isoflurane using a rodent circuit controller (RC2, Vetequip). After disinfecting the surgical area with povidone-iodine, a dorsal midline incision was made near the spine. The Con group bioconstruct was implanted on the left side of the spine, while either the Con-ColED group (15 mice) or the S-ColED group (15 mice) was implanted on the right side. The implanted bioconstructs were harvested during 2 and 4 weeks post-implantation for further analysis.

### Histology and immunochemistry examination

The harvested samples were fixed with 10% NBF (NBF, Leica Biosystem) for 24 h at room temperature for 24 h, then paraffinized and sectioned into 5 μm thick sections. Deparaffinized sections were stained with Hematoxylin and Eosin (H&E) staining and Masson's trichrome staining (MTS) was used to evaluate cellular morphology, ECM organization, and vessel formation after deparaffinization. Image J software was then utilized to quantify the remnant scaffold area and collagen area.

To further examine the collagen and vessel formation, immunofluorescence staining was performed on the deparaffinized sections. After the rehydration and subjection to antigen retrieval at 37°C for 30 min, samples were blocked with a serum-free blocking solution at room temperature for 1 h. Then, they were incubated with mouse anti-CD31 (5 μg/mL in DPBS; Invitrogen, USA), rabbit anti-alpha smooth muscle actinin (α-SMA) (5 μg/mL in DPBS; Invitrogen, USA), and rabbit anti-Col-III (5 μg/mL in DPBS; Invitrogen, USA primary antibodies at 4°C overnight. The secondary anti-mouse and anti-rabbit antibodies conjugated with Alexa Fluor 594 and 488 (1:50 in DPBS; Invitrogen, USA) were used to stain the tissues for 1 h. DAPI solution was used to counterstain the cell nuclei. The stained sections were visualized under the confocal microscope and ColIII+, CD31+, and vessel area were measured using Image J software.

### Statistical analysis

Statistical analyses were performed using SPSS software (SPSS, Inc., USA). One-way analysis of variance (ANOVA) with Tukey's honest significant difference (HSD) post-hoc test was used for comparisons among three or more groups, whereas Student's t-test was used for comparisons between two groups. Bonferroni correction was conducted before performing ANOVA. Values of ^*^p < 0.05, ^**^p < 0.01, and ^***^p < 0.001 were considered statistically significant.

## Supplementary Material

Supplementary figures.

## Figures and Tables

**Table 1:** List of primer sequences used in this study

Gene	Source	Primer sequence			GenBank number
		Left (5ʹ - 3ʹ)	Right (5ʹ - 3ʹ)		
*GAPDH*	*Homo sapiens*	TTTTGCGTCGCCAGCC		TTTTGCGTCGCCAGCC	NM_002046.7
*ACTB*	*Homo sapiens*	GCCTCGCCTTTGCCGAT		AGGTAGTCAGTCAGGTCCCG	NM_001101.5
*Col-I*	*Homo sapiens*	TGACGAGACCAAGAACTGCC		GCACCATCATTTCCACGAGC	NM_000088.4
*Col-III*	*Homo sapiens*	GGTCTCAGTGGAGAACGTGG		TCTGTCCACCAGTGTTTCCG	NM_000090.4
*Col-V*	*Homo sapiens*	CTTGGCCCAAAGAAAACCCG		CGAACGAATGGCATGCTGAG	NM_000093.5
*VEGF*	*Homo sapiens*	AGGCCAGCACATAGGAGAGA		ACGCGAGTCTGTGTTTTTGC	NM_001171623.1
*bFGF*	*Homo sapiens*	CCCAGAAAACCCGAGCGA		AGGAAGAAGCCCCCGTTTTT	NM_002006.5
*PIEZO-1*	*Homo sapiens*	ACTTTCCCATCAGCACTCGG		AGGGTGTAGAGCAACATGGC	NM_001142864.4
*TRPV2*	*Homo sapiens*	CTCACCTGAAAGCGGAGGTT		AGAGGCACCATCCTCATCCT	NM_016113.5
*TRPC6*	*Homo sapiens*	TTACGCTTCGCTACCACCAG		AGCAAAAGCCGGATGACTGA	NM_004621.6
*YAP*	*Homo sapiens*	CCCTCGTTTTGCCATGAACC		AATTCAGTCTGCCTGAGGGC	NM_001130145.3
*TAZ*	*Homo sapiens*	AGCCCTTTCTAACCTGGCTG		TGACTAATGCTGCTGCTGCT	NM_015472.6
*TEAD*	*Homo sapiens*	GTTTCTGTGGCAAAGCCCTG		TATCTGTCCACCAGCCGAGA	NM_021961.6
*WNT*	*Homo sapiens*	CCGCAACTATAAGAGGCGGT		AAGGTTCATGAGGAAGCGCA	NM_005430.4
*β-catenin*	*Homo sapiens*	GGCTACTCAAGCTGATTTGATGG		GCAGGAATGCCTCCAGACTT	NM_001098209.2
*G-protein*	*Homo sapiens*	ACGTGAGCTACCTGATGGC		ATGGCGTACATCTTGCCTGT	NM_001619.5
*FAK*	*Homo sapiens*	GCTCCCTTGCATCTTCCAGT		ATTGCAGCCCTTGTCCGTTA	NM_001352699.2
*PI3K*	*Homo sapiens*	GGGACCCGATGCGGTTAGAG		GGGCATCCTCCCAAGCATTA	NM_006218.4
*AKT*	*Homo sapiens*	CCAGGATCCATGGGTAGGAAC		CTCCTCCTCCTCCTGCTTCT	NM_001382430.1
*RHOA*	*Homo sapiens*	GTCCACGGTCTGGTCTTCAG		CAGCCATTGCTCAGGCAAC	NM_001664.4
*ROCK*	*Homo sapiens*	TGAAAGCCGCACTGATGGAT		AAGCAGCTCTCCTGGTTGAC	NM_005406.3
*MAPK1*	*Homo sapiens*	CCAGAGAACCCTGAGGGAGA		GGACCAGGGGTCAAGAACTG	NM_002745.5
*CD31*	*Homo sapiens*	CCAGGCATTTTGGACCAAGC		GGACAGCTTTCCGGACTTCA	NM_000442.5
*vWF*	*Homo sapiens*	CAGCCCCTGGGCTACATAAC		GAGGCATAGGGCATGGAGAC	NM_000552.5
*BAX*	*Homo sapiens*	TCAGGATGCGTCCACCAAGAAG		TGTGTCCACGGCGGCAATCATC	NM_138761.4
*BAD*	*Homo sapiens*	CCAACCTCTGGGCAGCACAGC		TTTGCCGCATCTGCGTTGCTGT	NM_032989.3
*AIFM1*	*Homo sapiens*	GGCTTCCTTGGTAGCGAACTGG		GTCCAGTTGCTGAGGTATTCGG	NM_004208.4
*CASP3*	*Homo sapiens*	GGAAGCGAATCAATGGACTCTGG		GCATCGACATCTGTACCAGACC	NM_004346.4
*CASP6*	*Homo sapiens*	AGGTGGATGCAGCCTCCGTTTA		ATGAGCCGTTCACAGTTTCCCG	NM_001226.4
					

*^GAPDH^* ^: glyceraldehyde-3-phosphate dehydrogenase; *ACTB*: beta actin; *Col*: collagen; *VEGF*: vascular endothelial growth factor; *bFGF*: basic fibroblast growth factor; *PIEZO-1*: Piezo type mechanosensitive ion channel component 1; *TRPV2*: transient receptor potential cation channel subfamily V member 2; *TRPC6*: transient receptor potential cation channel subfamily C member 6; *YAP*: Yes1 associated transcriptional regulator; *TAZ*: WW domain containing transcription regulator 1 (WWTR1); *TEAD*: TEA domain transcription factor; *b-catenin*: beta catemom; *G-protein*: G protein-coupled receptor (GPCR); *FAK*: focal adhesion kinase (protein tyrosine kinase 2, PTK2); *PI3K*: phosphoinositide 3-kinase; *AKT*: protein kinase B (PKB); *RhoA*: Ras homolog family member A; *ROCK*: Rho associated coiled-coil containing protein kinase; *MAPK1*: mitogen-activated protein kinase 1; *CD31*: platelet endothelial cell adhesion molecule (PECAM-1); *vWF*: von Willebrand factor; *BAX*: BCL2 associated X, apoptosis regulator; *BAD*: BCL2 associated agonist of cell death; *AIFM1*: apoptosis inducing factor mitochondria associated 1; *CASP3*: caspase 3.^

## Abbreviations

hGF: human gingival fibroblast
EA: EA.hy926 endothelial cell
Col-I, III, and V: collagen type I, III, and V
GdECM: Gingival decellularized extracellular matrix
EPA: eicosapentaenoic acid
DHA: docosahexaenoic acid
VEGF: vascular endothelial growth factor
PCR: polymerase chain reaction
VIN: vinculin
α-SMA: alpha smooth muscle actinin
H&E: Hematoxylin and Eosin
MTS: Masson's trichrome staining
c-bioprinter: comb-assisted bioprinter
NMS: nozzle moving speed
Con: control cell construct, normally bioprinted hGFs-loaded collagen type-I construct
Col bioink: hGFs loaded collagen type-I bioink
ColED: EPA/DHA, and hGFs loaded collagen type-I bioink
S-ColED: c-bioprinted EPA/DHA, and hGFs-loaded collagen type-I construct
N-ColED: normally bioprinted EPA/DHA, and hGFs-loaded collagen type-I construct
