# Supplementary material for: Mechanotransduction-enhanced bioconstructs fabricated using a bioink comprising collagen and omega-3 fatty acids for gingival tissue regeneration
Source: Theranostics. 2025 May 25;15(13):6476–96. doi: 10.7150/thno.114503 (PMC12160015; doi:10.7150/thno.114503)
Supplement: Supplementary file 1 — Supplementary figures. [file thnov15p6476s1.pdf]

Supplementary Material

Mechanotransduction-enhanced bioconstructs fabricated using a bioink comprising collagen and omega-3 fatty acids for gingival tissue regeneration

GaEun Heo[a,†], Hoon Noh[b,†], Dogeon Yoon[c,†], SooJung Chae[a], Hanjun Hwangbo[a] , Ji Hye Park[c], Won Hee Lim[b,*], WonJin Kim[a,d,**], GeunHyung Kim[a,e,***]

[a]Department of Precision Medicine, Sungkyunkwan University School of Medicine (SKKU-SOM), Suwon 16419, Republic of Korea

[b]Department of Orthodontics, School of Dentistry and Dental Research Institute, Seoul National University, Seoul, Republic of Korea

[c]Burn Institute, Hangang Sacred Heart Hospital, College of Medicine, Hallym University, Seoul, Republic of Korea

[d]Department of Convergence Pharmaceutical Science, Korea University, Sejong, 30019, Republic of Korea

[e]Biomedical Institute for Convergence at SKKU (BICS), Sungkyunkwan University, Suwon 16419, Republic of Korea

[†]These authors contributed equally to this work.

Corresponding authors:

[*]Prof. Won Hee Lim, E-mail: whlim@snu.ac.kr

[**]Prof. WonJin Kim, E-mail: joshbass@korea.ac.kr

[***]Prof. GeunHyung Kim, E-mail: gkimbme@skku.edu

Figures

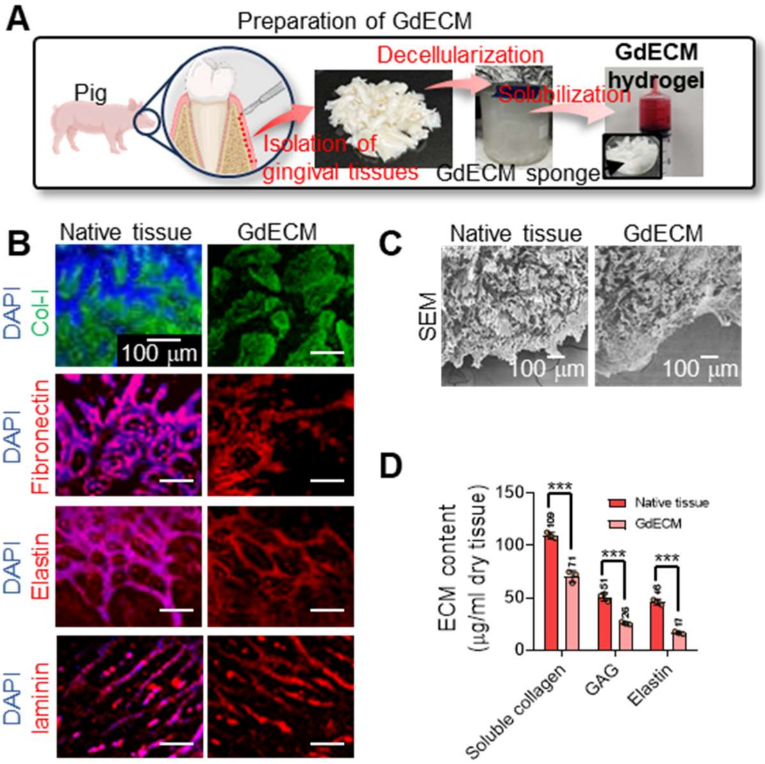

Figure S1. Preparation and characterization of GdECM. (A) Schematics demonstrating the preparation of porcine-derived GdECM. (B) Immunofluorescence images including Col-I, fibronectin, elastin, and laminin and (C) SEM images for the native gingival tissue and GdECM. (D) Quantitatively estimated ECM contents after conducting the decellularization procedures (n = 3). All data are mean ± SD (***$p < 0.001$, analyzed using Student's t-test).

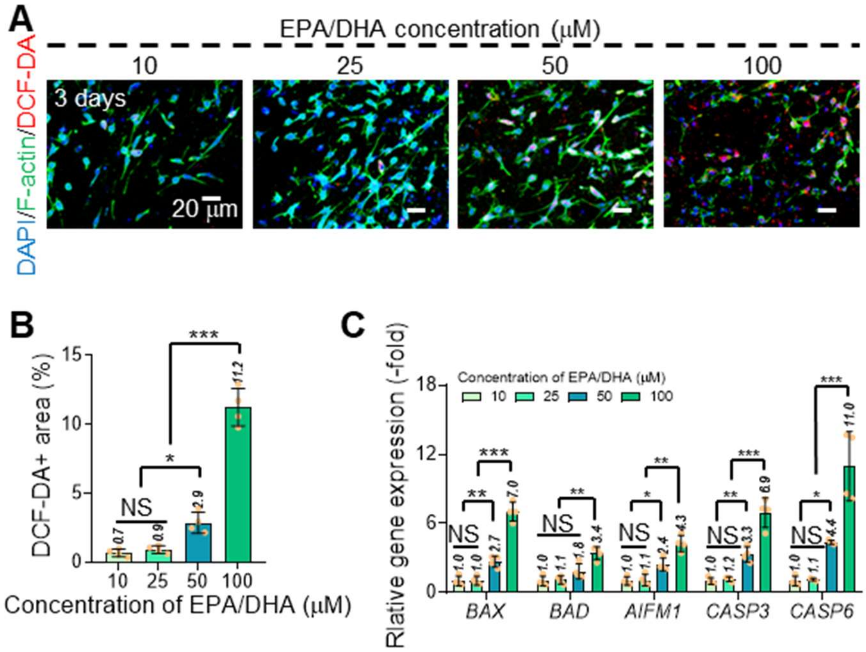

Figure S2. Analysis for the cellular ROS deposition and apoptosis. (A) Immunofluorescence images of DCF-DA and (B) quantitatively estimated DCF-DA+ areas for the cells within the bioinks containing different concentrations of EPA/DHA at 3 d (10, 25, 50, and 100 μM) (n = 4). (C) Expression of cellular apoptosis-related genes at 3 d (n = 4). All data are mean ± SD (NS = no significance, *p < 0.05, **p < 0.01, ***p < 0.001, analyzed using ANOVA with Tukey's HSD post-hoc test).

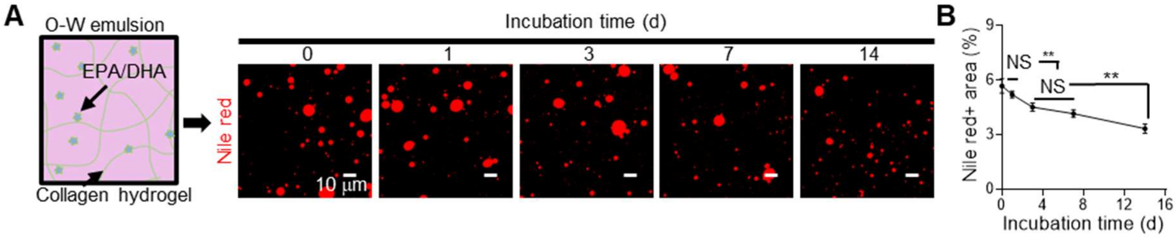

Figure S3. Characterization of EPA/DHA-incorporated bioink. (A) Nile red images demonstrating the oil in water emulsion (O-W emulsion) of EPA/DHA within the collagen-based bioink and (B) quantitatively measured Nile red+ area at 0, 1, 3, 7, and 14 d of culture (n = 4). All data are mean ± SD (NS = no significance, **$p < 0.01$, analyzed using ANOVA with Tukey's HSD post-hoc test).

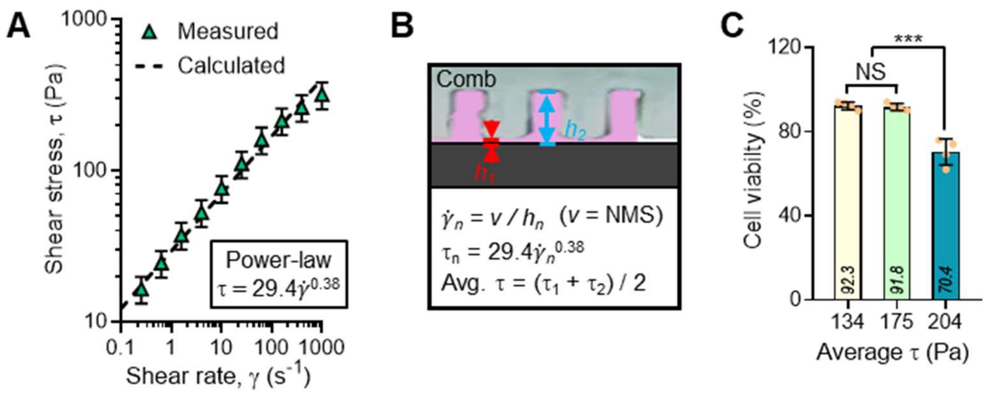

Figure S4. Analysis for the cell viability under diverse shear stress conditions. (A) Shear rate versus shear stress curves of the ColED bioink. (B) Schematic illustration describing the average shear stress (Avg. τ) applied to the bioinks under different NMS conditions. (C) Cell viability of bioprinted hGFs under different Avg. τ values at 4 hours post-printing (n = 4). All data are mean ± SD (NS = no significance***p < 0.001, analyzed using ANOVA with Tukey's HSD post-hoc test).

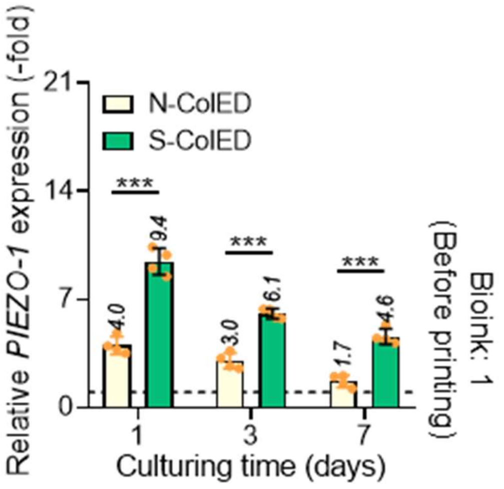

Figure S5. Evaluation of comb-assisted bioprinting on the biological responses of hGFs. (F) Expression of *PIEZO-1* gene after 1, 3, and 7 d of culture (n = 4). All data are mean ± SD (***$p < 0.001$, analyzed using Student's t-test).

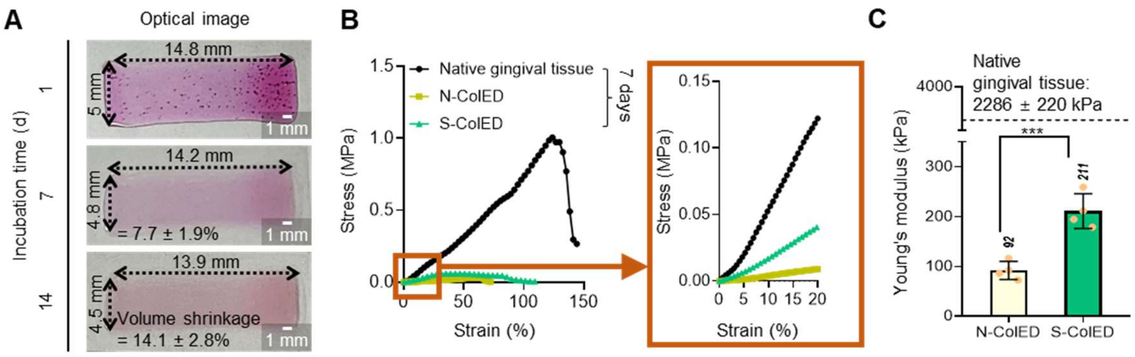

Figure S6. Characterization of c-bioprinted gingival tissue. (A) Optical images of c-bioprinted structures after 1, 7, and 14 d of incubation. (B) Stress-strain (S-S) curves of porcine-derived native gingival tissue and bioprinted gingival tissues (N-ColED and S-ColED) at 7 d. (C) Quantitatively estimated Young's modulus of N-ColED and S-ColED using linear region of the S-S curves (n = 4). All data are mean ± SD (***p < 0.001, analyzed using Student's t-test).
